# Gender and caste inequalities in primary healthcare usage by under-5 children in rural Nepal: an iterative qualitative study into provider perspectives and the potential role of implicit bias

**DOI:** 10.1136/bmjopen-2022-069060

**Published:** 2023-06-26

**Authors:** Saugat Joshi, Alisha Karki, Simon Rushton, Bikash Koirala, Srijana Basnet, Barsha Rijal, Jiban Karki, Gerda Pohl, Manish Baidya, Tim Chater, Dan Green, Andrew Lee

**Affiliations:** 1 PHASE Nepal, Kathmandu, Nepal; 2 Department of Politics and International Relations, The University of Sheffield, Sheffield, UK; 3 Department of International Public Health, Liverpool School of Tropical Medicine, Liverpool, UK; 4 Liverpool Clinical Trials Centre, University of Liverpool, Liverpool, UK; 5 College of Health and Life Sciences, Aston University, Birmingham, UK; 6 School of Health and Related Research, The University of Sheffield, Sheffield, UK

**Keywords:** Health policy, Quality in health care, International health services, EDUCATION & TRAINING (see Medical Education & Training)

## Abstract

**Objective:**

This study explored provider perspectives on: (1) why inequalities in health service usage persist; and (2) their knowledge and understanding of the role of patient experience and implicit bias (also referred to as unconscious bias).

**Design:**

A three stage, iterative qualitative study was conducted involving two rounds of in-depth interviews and a training session with healthcare staff. Interview transcripts were analysed using a reflexive thematic approach in relation to the study’s aims.

**Setting:**

Participants were recruited from rural hill districts (Mugu, Humla, Bajura, Gorkha and Sindhupalchok) of Nepal.

**Participants:**

Clinical staff from 22 rural health posts.

**Results:**

Healthcare providers had high levels of understanding of the cultural, educational and socioeconomic factors behind inequalities in healthcare usage in their communities. However, there was less knowledge and understanding of the role of patient experience—and no recognition at all of the concept of implicit bias.

**Conclusion:**

It is highly likely that implicit bias affects provider behaviours in Nepal, just as it does in other countries. However, there is currently not a culture of thinking about the patient experience and how that might impact on future usage of health services. Implicit bias training for health students and workers would help create greater awareness of unintended discriminatory behaviours. This in turn may play a part in improving patient experience and future healthcare usage, particularly among disadvantaged groups.

**Strengths and limitations of this study**

- Existing research on health service usage in Nepal has been from the patient perspective: this study examines service providers’ perspectives on the factors that cause inequalities in health service usage.
- The study produced strong evidence that healthcare providers have a good understanding of the social and economic factors that determine service usage, and can point to instances of discriminatory treatment.
- To our knowledge, this is one of the few studies that has investigated the possibility of introducing implicit bias training for health workers in Nepal.
- Participants in the study were health post staff employed by a Nepali non-governmental organisation in selected districts, and do not necessarily reflect the perspectives of all healthcare providers in all areas of the country.
- This study did not examine the experiences of patients, parents or caregivers who receive services: this would be an important avenue for future research.

## Introduction

The world has made substantial progress in reducing childhood mortality and child survival over the past three decades. Globally, the under-5 mortality rate has decreased by 60%, from 93 deaths per 1000 live births in 1990 to 37 in 2020.^1^ Nevertheless, around 5 million children still die each year, largely from causes that are preventable.^1^ The health of young children is especially susceptible to socioeconomic inequalities, which have led to significant differences in childhood morbidity and mortality between and within countries.^2^

Among the countries of South Asia, Nepal has the highest reported prevalence of under-5 mortality over the past 15 years.^3^ In recognition of this, child health is one of the priority programmes of the government, which has targeted reducing under-5 mortality to 28 per 1000 live births and neonatal mortality to 12 per 1000 live births by 2030.^4^ As a result of this prioritisation, significant improvements have been made in child health services and outcomes over recent years.^5^ Despite this, socioeconomic inequalities remain an obstacle to tackling Nepal’s continuing high rates of child illness and death.^6^ In addition to continuing inequalities in the socioeconomic determinants of health, health services are still not effectively reaching all individuals, most commonly those from the low castes, ethnic minorities, people living in remote areas and other marginalised populations.^7^ Deeply-rooted societal, gender, caste/ethnic, regional and wealth inequities have made it difficult to address this situation.

Healthcare usage by children under-5 depends on the health-seeking behaviours of their parents and caregivers. As seen in studies elsewhere in South Asia, parents and caregivers do not always appropriately seek healthcare services for childhood illness for a variety of reasons, including low levels of parental education,^8–10^ a preference for traditional healers^11^ and living long distances from a health post.^12^ Health-seeking patterns for children have also been found to be highly gendered in Nepal,^13^ as in other Asian countries.^14 15^ Delays in care-seeking can lead to illnesses becoming more severe, or in some cases mean that the child does not receive treatment at all.

Stigma is one of the causes of health inequalities,^16^ and discriminatory attitudes and practices among health service providers can discourage healthcare usage by certain groups. For example, although caste-based discrimination is illegal in Nepal, continuing cultural belief in the ‘untouchability’ of Dalits has been found to limit access to healthcare services.^16^ When patients do present at a health facility, studies in other settings have shown that patients’ experience can significantly affect future usage: if people are dissatisfied with services, or feel discriminated against when using them, they are less likely to return.^17–19^ One study conducted in Nepal found that only one-third (35.9%) of respondents reported receiving good communication from health service providers,^20^ suggesting that shortcomings in patient experience are widespread. Ensuring a good patient experience for all is therefore an important element of attempts to promote health service usage among marginalised groups.^21^

However, this is an under-researched area in Nepal. Relatively few studies have examined the barriers to healthcare usage for under-5s in Nepal. Those studies that do exist have examined socioeconomic factors affecting the likelihood of people seeking health services from the perspective of patients.^10 11^ While recognising the importance of this patient perspective, in this study we seek to complement this previous work by: (1) adopting a service provider-focused perspective, to understand their perceptions on the reasons for unequal patterns of healthcare usage; and (2) to explore service providers’ perspectives on patient experience and how they think this may relate to service usage by different groups.

This qualitative study builds on the findings of a previous quantitative study in which paper-based patient records from 23 rural health posts were digitised and analysed to understand inequalities in health service usage, diagnosis and treatment for under-5 children.^22^ The data revealed significant differences by gender, caste and geography. For example, we found that children in more remote districts took longer to present at a health facility after the onset of symptoms than children in less remote districts. When children were brought to a health facility, those from underprivileged groups (eg, Madhesi and Dalit) were less likely to be given a correct diagnosis of pneumonia, while male children were more likely to receive a correct diagnosis than female children, suggesting inequalities in the quality of treatment received. While this previous study revealed these inequalities, it did not explain *why* those differences occurred. In the follow-up study reported here, we conducted in-depth interviews (IDIs) with service providers from those same health posts to explore their perceptions as to why these differences exist in the communities in which they work, and subsequently designed and implemented training sessions based on role-play scenarios to increase their knowledge and understanding of patient experience.

## Methods

### Study design

We applied a qualitative approach in this study, employing IDIs with health workers from rural health posts to explore inequalities in service usage, focusing on providers’ perceptions of the causal mechanisms within the rural communities in which they work. After the initial round of analysis identified notable gaps in the data around the potential effect of service providers’ own attitudes and practices, we designed and implemented training and subsequently conducted a second round of IDIs to explore interviewees’ awareness and understanding of implicit bias (also referred to as unconscious bias).

### Setting

In Nepal, each ward (the smallest political unit) has a government-run health post and community health unit providing primary healthcare and (where necessary) referral on to secondary health facilities. In some cases (as in this study) those facilities are additionally supported by staff employed by non-governmental organisations (NGOs). The participants in this study were current clinical staff members, employed by a Nepali NGO, drawn from 22 of the 23 rural health posts that had been part of the preceding quantitative study (for logistical reasons, it was not possible to interview staff from one health post). We report this study in accordance with the Standards for Reporting Qualitative Research checklist.

### Research participants

We initially conducted IDIs with one health service provider currently working in each of 22 health posts spread across four provinces of Nepal (Bagmati, Gandaki, Karnali and Sudurpaschim provinces), covering the rural hill districts of Gorkha, Sindhupalchok, Mugu, Humla and Bajura. These particular health posts were selected for both the original quantitative study and this follow-up study as they are currently supported by the NGO partner organisation involved in this study, a national NGO employing a total of 78 staff.

Within each health post, we purposively selected the most experienced member of NGO staff (measured in terms of years of service) in order to get as informed an account as possible of the possible reasons for inequalities in healthcare usage. The years of experience ranged from 2 to 14 years. Interviewees included auxiliary nursing midwives, staff nurses and paramedics. The majority of interviewees had experience of having worked in multiple rural health posts. Nineteen of the interviewees were women and three men, with ages ranging from 25 to 35 years. Further characteristics of the interviewees and the associated participant codes are presented in table 1.

**Table 1:** Characteristics of interviewees

Participant code	Ethnicity	Education qualification
KII_F_01	Janajati	Intermediate
KII_F_02	Brahmin/Chhetri	Staff nurse
KII_F_03	Brahmin/Chhetri	Intermediate
KII_F_04	Brahmin/Chhetri	ANM
KII_F_05	Brahmin/Chettri	ANM
KII_F_06	Dalit	ANM
KII_M_07	Brahmin/Chhetri	ANM
KII_F_08	Dalit	ANM
KII_F_09	Brahmin/Chhetri	Staff nurse
KII_M_10	Brahmin/Chhetri	Health assistant
KII_F_11	Dalit	Health assistant
KII_F_12	Brahmin/Chhetri	ANM
KII_F_13	Brahmin/Chhetri	ANM
KII_F_14	Janajati	ANM
KII_F_15	Brahmin/Chhetri	ANM
KII_F_16	Janajati	Staff nurse
KII_F_17	Janajati	ANM/intermediate pass
KII_M_18	Brahmin/Chhetri	Health assistant
KII_F_19	Dalit	ANM
KII_F_20	Brahmin/Chhetri	ANM/10+2
KII_F_21	Brahmin/Chhetri	ANM/10+2
KII_F_22	Janajati	ANM

ANM, auxiliary nursing midwives.

### Data collection and management

Data collection was via a three-stage iterative process in which the research plans were adapted at each stage in response to initial findings.

The initial round of interviews was undertaken during a week of training events attended by all health staff of the project’s NGO partner (which was why only NGO staff, and not government staff, participated). Prior to the IDIs, the results of the preceding quantitative study (ie, the data on gender, geographic and caste inequalities in these specific health posts) were presented to all of the NGO’s staff. The selected participants were then interviewed individually to ascertain their perspectives on *why* these inequalities persist.

The semi-structured interview guide (see online supplemental file 1) was developed based on our previous quantitative study, expert input from medical practitioners, major themes from existing literature and discussion between the members of the research team. The interview guide was initially developed in English and then translated into Nepali. We conducted four interviews as a pre-test and made necessary changes to the interview schedule to improve the clarity of questions. We then conducted the 18 remaining IDIs.

10.1136/bmjopen-2022-069060.supp1 Supplementary data

A rapid analysis of the emerging findings from this first round of 22 interviews was discussed by the members of the research team, and omissions/notable gaps in the data were considered,^23^ taking into account the published evidence on health service usage (in Nepal and elsewhere). During this analysis, the potential role of negative patient experience and implicit bias leading to suboptimal patient experience—even where providers themselves had the best of intentions—was identified as a notable ‘silence’ in the data.

In the second stage, in response to these emerging findings, a training session on patient experience was designed, drawing on input from team members who had been involved in similar training sessions in other countries. The training session was held 2 days after the final interview. It included a role-play session conducted in pairs in which half of the participants were asked to play the role of a patient bringing a baby to a health post, and the other half to play the role of the service provider. After the role-play consultation was complete, the participants swapped roles. This was followed by a guided debrief discussion in which patient experience was discussed, first in pairs and later as a whole group, and participants’ perspectives on necessary elements of a positive patient experience were collated. This session was designed to prompt reflection among participants on patient experience.

In the third stage (2 weeks after the training event had finished and participants had returned to their health posts), a new interview guide was developed focusing on the themes of patient experience and participants’ knowledge and understanding of implicit bias. Based on this guide, a second round of follow-up interviews was conducted, via telephone, with all 22 study participants.

All interviews took place in April and May 2022, carried out by five experienced qualitative researchers (AK, SJ, BR, SB and BK). The training session additionally involved facilitation by JK, GP and SR. We obtained written consent from participants. Both the face-to-face interviews and the telephone interviews were audio recorded with the participants’ consent. The average length of the interviews was 25 min.

We first transcribed the recordings verbatim into Nepali, and then translated these transcriptions into English. All personal identifiers were replaced with unique codes (see table 1). Seven of the interview transcripts were back translated to ensure that no meaning was lost during the translation process.

### Data analysis methods

Two researchers (AK and SJ) coded the interview transcripts using a reflexive thematic approach in NVivo V.12 (V.12 Pro, QRS International) qualitative data management software.^24^ The researchers read the full transcripts before starting the coding process. Following this, we organised all the text from the interviews by coding and grouping them into appropriate themes, supported by discussion among the members of the research team. During the process, we developed initial sets of codes while thoroughly reading the text followed by combining and modifying them, thus developing a more refined codebook. Finally, we grouped the codes into major thematic categories and the whole research team finalised the codebook for the data analysis process.

### Patient and public involvement

None.

### Findings

#### First round of interviews: provider explanations for differential usage of health services

In this section, we present the explanations given by service providers in the first round of interviews for the observed differences in health service usage in the areas in which they work according to (1) caste and (2) gender.

#### Caste

##### Lack of knowledge and education

Most interviewees (n=16) indicated that, in their experience, people from the lower castes lack awareness, knowledge and education on the causes, symptoms and treatments for diseases.

When a Brahmin child gets sick, mostly the parents bring them to the health post as soon as possible. But people belonging to the Dalit caste are not educated or aware enough to make such informed decisions. KII_F_08

People of lower caste are uneducated and are unaware of their health. Janajati people make the children drink local alcohol to treat their condition. KII_F_22

##### Economic factors

The majority of interviewees (n=16) noted that, in comparison to people from other castes, Dalits tend to be poorer.

The economic situation of Dalits is also poor compared to other groups, which is why they tend to seek household remedies to cure the illnesses of their children. KII_F_08

Although services at health posts are provided free of charge, interviewees explained that economic deprivation could nevertheless affect service usage: those from lower castes have less flexibility with their time, often having to work long hours in their own fields and/or for other people to make a living. As a result, they are not able to visit the health post without incurring financial losses, and thus tend not to bring their children for treatment in the early stage of illness.

##### Preferential use of traditional healers

Most interviewees (n=18) reported that traditional healers’ services are often used at the community level, either because they are preferred by patients, or because they are more conveniently located.

In rural areas, faith healers are still the first choice for the majority of people. People who live near health posts try to bring their children as soon as possible, but people who live far from the village centre first try traditional remedies and consume medicinal herbs. KII_F_08

People who live far from the health post prefer to take their children to a faith healer to treat their condition. They only come to a health facility if they are referred by the faith healer, or if the faith healer is not able to treat their condition. KII_F_06

Some interviewees (n=8) reported that older people and those who are less educated (often from lower castes) are particularly likely to believe in Gods and Goddesses and prefer to participate in rituals and make offerings in case of illness. These groups were seen as more likely to take their children to traditional healers, and health education efforts were seen as having had little impact so far:

We are involved in conducting household programs to raise the awareness level and educate people about the importance of their children’s health. We ask them to bring their children [to the health post] as soon as they get sick. However, people in the village are still following outdated practices that were taught by their ancestors. KII_F_10

##### Deep rooted (and internalised) stigma

The deep-rooted stigma and discrimination against Dalits in the community was seen as reducing their willingness to bring their children to the health post, resulting in self-exclusion from services. When they do bring their children to the health post, interviewees reported that other parents (eg, those from the Brahmin/Chhetri caste) insist that their children should be treated first. Some interviewees (n=5) reported that many Dalits themselves believe that it is a sin if they touch an upper caste person, so even when they do go to the health post, they sit in a corner, away from other waiting patients. One of our participants said that:

When we demonstrated how ‘jaulo’ [a kind of porridge] is made, we invited all the people from different castes. When they came, all the so-called upper caste people sat on one side and the lower-class people sat on the other side. While eating food, they did not touch each other. It’s not like people set a rule about such things, but the culture was such that a Dalit person believes that it is not nice to touch a person of upper caste. KII_F_06

#### Gender

##### Differential attitudes towards male and female children

Providers believed that parents are more likely to take sons to health facilities quickly when they are sick, and to spend more money on their care than they do for their daughters. They reported that parents of daughters are less likely to seek care outside of home and, even when seeking care, they are more likely to go to an informal or traditional healer. Even mothers were seen as being more vigilant about the health of their sons.

There was a woman in Bajura district who had three daughters. One of her daughters had severe pneumonia, but she did not bring her to the health post. She said that it would not matter if one daughter dies because she was still of childbearing age and could give birth to another one if this one dies. KII_F_03

In the community I worked in, there was a woman who had a son after four daughters. She didn’t bring any of her daughters when they got ill. But she used to get worried and concerned and brought her son if he suffered even a minor cold. She used to say that after waiting and praying for a long time, they were able to have a son. She brought her son as if it was a very serious case, but it was normal. KII_F_02

Service providers attributed these differences in health-seeking behaviours to deeply-rooted cultural norms around the relative value of male and female children, and their expected future roles and contribution to the family.

A girl child doesn’t even get a naming ceremony. A mother gives a random name based on what she likes. KII_F_08

People say that a daughter is not going to earn and she will not achieve anything big in life, so they tend to deprive a female child of education as well as not seeking health services. They say that there is no point in educating a daughter since she will get married after a certain age. KII_F_06

People believe that a male child can carry the legacy of their parents forward. Sometimes, when we are not able to treat their daughters, we refer them to a hospital. When we do so, they ask us if we can treat her or not. When we say no, then they don’t think it is worth taking her to the hospital. Even if the daughter dies, the mother does not feel guilty. KII_F_06

### Second round of interviews: interviewees’ knowledge and understanding of patient experience and implicit bias

As described above, the first round of interviews revealed that interviewees had a very good awareness of the ways in which caste and gender could affect health service usage in the communities in which they were working. They did not, however, identify the potential role of provider-side attitudes and behaviours. The training session on patient experience and the follow-up interviews explored these themes in depth. In the second round of interviews, we examined interviewees’ experience of discriminatory behaviours, and their knowledge and understanding of the potential impact of implicit bias on their own treatment of patients.

#### Experiences of unequal patient experience

When asked about the respective experiences that different groups may have when visiting a health post, many interviewees (n=14) were able to give examples of having witnessed colleagues not treating every patient equally.

It is not so common but, in some cases, health personnel tell a Dalit patient to keep their distance and ask them to do things on their own, just to avoid touching them as they are considered untouchables. KII_F_08

When I was working in a remote area of Gorkha district, Dalit children were not allowed to sit with other patients and they received their check-up only after Brahmin and Chhetri children had been seen. KII_F_16

People of certain castes were not given the respect they deserve and staff [government health workers] mostly tried to ignore them as much as possible, just to avoid touching them. When we [NGO staff] tried to touch them to treat them, we were told to not to touch such patients and to avoid physical contact. KII_F_09

As most of the [government] staff working in the health facility are local, they treat patients based on their caste/ethnicity. People who are from the upper castes are treated with utmost care and respect and those who belong to lower castes are neglected and, sometimes, mistreated as well. KII_F_09

In all cases, such discriminatory treatment was attributed to others—and in all cases to government health staff rather than staff of the NGO (we acknowledge here a strong possibility of self-serving bias).

Although, when asked in the training session and interviews, participants agreed with the logic that patient experience could have an impact on whether or not parents chose to bring their children back to the health post in case of future illness, most indicated that there was no effective feedback mechanism in the health facilities where they work to capture patients’ (dis)satisfaction with their experience.

We know that from their body language. We don’t have any specific mechanism, but whenever a patient tells us then we get to know about their experiences. KII_F_02

Furthermore, interviewees said that they had not previously received training relating to patient experience. Reflecting on the role play-based training session provided to participants by the research team, one reflected that.

Previously, we used to see things from the side of a health service provider, but this roleplay session made us realise that we need to treat patients with patience. We do not have much patience as we have to see many patients in a day. I think [this training] will definitely support us in our work in the future. KII_F_05

#### Knowledge and understanding of implicit bias

Although discriminatory treatment was recognised (and always attributed to others), all interviewees (n=22) reported that they had never heard about the concepts of unconscious or implicit bias, nor received any training on it. For example,

I don’t think I have heard about such a term. We don’t intentionally try to be biased towards a particular individual. However, there might be some cases that we are not aware of. KII_F_09

I don’t think I have heard or had such an experience about implicit bias in my professional life. KII_F_07

I have not received any specific training on that. In other training that we received, we are only taught about treating the patient right but we are not aware of the bias related training that you mentioned. KII_F_04

## Discussion

We found that rural healthcare providers had a high level of awareness and understanding of the cultural, educational and socioeconomic factors behind inequalities in healthcare usage. The lack of awareness, knowledge and education among Dalits and other low castes, their preference for traditional healers, deep-rooted and internalised stigma, and differential parental attitudes towards male and female children, were all commonly identified, and were in accordance with findings in the existing literature from Nepal^6 7 10 11 13 25 26^ and elsewhere in South Asia.^8–10 12 15 16 27 28^

However, participants tended to have far low levels of awareness, understanding and training on the ways in which patient experience might adversely impact service usage. Although many respondents reported witnessing discriminatory behaviour by other health service providers, they generally believed that they treat everyone equally in their own practice (again, we acknowledge the risk of self-serving bias here). Studies from other countries have demonstrated that poor (and especially discriminatory) patient experience can deter future health service usage, as well as affect treatment adherence.^17–19 21 29–31^ However, our findings suggest that in Nepal there is not currently a culture of thinking about patient experience. Neither is there awareness among frontline health staff of the concept of implicit bias.

Most previous studies on the role of implicit bias have taken place in the Global North. For example, a narrative review indicated that healthcare providers in the USA show implicit negative attitudes and stereotypes about several stigmatised groups.^32^ A systematic review found consistent evidence that health professionals in the United States have implicit bias, leading to preferential treatment of white patients over people of colour.^33^ These studies suggest that being a child does not protect one from being on the receiving end of implicit bias. Although we did not use methods that could have demonstrated attitudes or behaviours associated with implicit bias among our participants, the existing literature from elsewhere suggests that it is extremely unlikely that our interviewees are completely free from such biases. The fact that implicit bias is frequently unrecognised by those who hold such biases is well known. Numerous studies have shown that self-reporting of implicit bias is not reliable as it is challenging for individuals to critically evaluate their own thoughts.^34 35^ Such biases exist in everyone, even well-intentioned healthcare workers (and also, of course, the members of this research team). Evidence indicates that healthcare professionals have the same levels of implicit bias as the general public.^36^

Healthcare providers’ awareness of implicit bias is not well documented in the context of Nepal and our literature review revealed very few previous studies in Nepal gave this topic even a passing mention^37^ (for an exception see^38^). However, it is evident that implicit bias is significantly related to patient-provider interactions, treatment decisions and patient health outcomes in all contexts,^33^ and certainly will be in Nepal. All of this leads us to suspect that bias among our participants is currently unrecognised rather than absent.

This study has revealed a need for further research on implicit bias in healthcare settings in Nepal. While the literature from other countries suggests that it is highly likely that implicit bias impacts on patient treatment in Nepal, it is important to understand the extent, consequences and drivers of this bias in the particular social context of Nepal—as well as to explore effective ways to counter this.

Our findings also have implications for practice as implicit bias has the potential to affect both treatment outcomes, and also whether parents (especially those from disadvantaged groups) bring their children to health facilities for treatment in future. Studies from other countries have shown that the provision of training to increase the awareness of individuals about their own biases can have a positive impact, equipping them to better manage and account for these biases in future.^39^ Many different approaches to such training are available. To take one example, the Implicit Association Test (IAT), created in 1998, is the most widely used computerised online tool for measuring and bringing awareness of implicit bias.^40^ Neither the IAT nor any of the myriad other forms of training and awareness-raising about implicit bias have, to our knowledge, been widely used in Nepal—including among healthcare workers. Although training cannot by itself completely remove bias, confronting health service providers with evidence of their biases and providing training on how to counter these biases is a necessary first step.^32 40^ We would recommend that Nepalese health/medical schools, the government of Nepal and other employers of health service providers in the country investigate the possibility of introducing implicit bias training.

## Conclusion

The impact that provider-side factors may have on health service usage in Nepal has not been widely studied, and this is, to our knowledge, the first paper to explore service providers’ perspectives on the potential roles of patient experience and implicit bias in deterring health post usage for young children.

In some ways, our findings make uncomfortable reading for the NGO partner that participated in this study. However, we would argue that there is a more positive way of interpreting these findings. Given what is known about the difficulties of relying on self-reported biases, implicit bias is likely to be an issue across all health workers in Nepal and not specific to this particular NGO. This NGO is the first in Nepal, to our knowledge, to properly investigate this issue. Following on from the training provided in patient experience, the next step in this process—in line with our recommendation above—would be for the NGO to pursue the wider use of validated implicit bias training with staff, in order to provide them with the practical awareness and skills to recognise and mitigate their own biases as well as their unintentional involvement in the perpetuation of discrimination.

More widely in Nepal, implicit bias training (in health/medical schools and for government and non-government health staff) would be a first step in creating greater awareness about unintended discriminatory behaviours, improving patient experience as well as future healthcare usage among disadvantaged groups. Policymakers and curriculum designers should introduce such training in order to minimise the impact of implicit bias in order to improve patient experience and, in turn, encourage greater usage of health facilities among marginalised groups.

## Supplementary Material

Reviewer comments

Author's manuscript

## Data Availability

Data are available upon reasonable request. Anonymised data are available upon request.
