# Supplementary material for: Gender and caste inequalities in primary healthcare usage by under-5 children in rural Nepal: an iterative qualitative study into provider perspectives and the potential role of implicit bias
Source: BMJ Open. 2023 Jun 26;13(6):e069060. doi: 10.1136/bmjopen-2022-069060 (PMC10410982; doi:10.1136/bmjopen-2022-069060)
Supplement: Supplementary data [file bmjopen-2022-069060supp001.pdf]

Gender and caste inequalities in primary healthcare utilization by under-5 children in rural Nepal: an iterative qualitative study into provider perspectives and the potential role of implicit bias

Interview guides

1. First round interviews

1. Could you please introduce yourself?

कृपया तपाइको परिचय दिनुहोस् ।

[Name, age, designation, place of work, qualification/education, experience in years (in rural areas), overall work experience)

[नाम , उमेर , पद , शिक्षा/योग्यता , अनुभव(वर्षमा) (ग्रामीण क्षेत्रमा), समग्र कार्य अनुभव]

2. Health seeking behavior (In terms of Gender, Ethnicity and Geography)

स्वास्थ्य सेवा लिन स्वास्थ्य संस्था आउने बानी/ प्रचलन (लिङ्ग, जाती , भूगोल को आधारमा)

2.1 How do people access the health post when they are unwell? What determines whether parents do or do not bring their child, and how quickly?

मानिसहरू अस्वस्थ (बिरामी) हुँदा कसरी स्वास्थ्य संस्था सम्म आइपुग्छन् ? अभिभावकले बच्चालाई स्वास्थ्य संस्था ल्याउने वा नल्याउने कुरा के ले निर्धारण गर्छ र कत्तिको छिटो ल्याउँछन् ?

2.2 Have you noticed any differences between different groups regarding how quickly they bring their child for treatment?

स्वास्थ्य संस्थामा बिरामी बच्चालाई उपचार गर्ने ल्याउने क्रममा कति छिटो ल्याइन्छ भन्नेमा फरक फरक समूहमा भिन्नता पाउनु भएको छ ?

2.3 The research data reflects differences between some groups? How do you think this can be explained?

अनुसन्धानको तथ्याङ्क (डाटा) हरूमा फरक फरक समूहमा भिन्नता देखिएको छ ? यसलाई कसरी व्याख्या गर्न / बुझ्न सकिन्छ ?

2.4 [Possible solutions for discussed issues/ problems]

छलफल भएका समस्याहरूको समाधान के हुन सक्छ होला भनेर सोध्नुहोस्

3. Discrimination during diagnosis and treatment (in terms of Sex, Ethnicity, Geography)

स्वास्थ्य संस्थामा निदान तथा उपचार गर्ने क्रममा हुने भेदभाव (लिङ्ग, जाती , भूगोल को आधारमा)

3.1 What kind of different groups visit this health post/facility?

यो (तपाईँले काम गर्ने) स्वास्थ्य संस्थामा कुन कुन समूह वा समुदायका मानिसहरू आउने गर्छन् ?

3.2 Can you tell us a bit about the skilled health workers in this facility?

यो स्वास्थ्य संस्थामा दक्ष / तालिम प्राप्त स्वास्थ्यकर्मीहरूको बारेमा केही बताइदिनुहुन्छ ?

3.3 Do you think some health workers treat different groups differently?

तपाईँको बिचारमा केही स्वास्थ्यकर्मीहरुले फरक फरक समूह /समुदायका मानिसलाई लाई फरक किसिमले व्यवहार गर्छन् ?

3.4 The research data reflects difference in diagnosis and treatment among different groups? How do you think this can be explained?

अनुसन्धानको तथ्याङ्क (डाटा) ले निदान र उपचारमा फरक फरक समूह बिचमा भिन्नता भएको पाइएको छ | यसलाई कसरी लिनुहुन्छ (कसरी व्याख्या गर्न/बुझ्न सकिन्छ)?

3.5 Knowing that there are differences, how do you think this can be improved?

उपचार र निदानका क्रममा भिन्नताहरू छन् भन्ने जानिसक्दा , तपाइको बिचारमा यसलाई सुधार गर्न के गर्नु पर्छ होला ?

2. Second round interviews

1. Have you previously received any training about patient experience (before the recent training session)?

स्वास्थ्यकर्मीले दिएको सेवाप्रति बिरामीको अनुभबबारे तपाइले कुनै तालिम लिनु भएको छ?

1.1 Could you provide us any example where you have experienced implicit bias in terms of providing health service?
तपाइले दिनुभएको निष्पक्ष सेवाप्रति कुनै बिरामीले नराम्रो अनुभब गर्नुभएको कुनै उदाहरण दिनसक्नु हुन्छ?

1.2 What was your experience of the roleplay session we conducted during our training workshop? What do you think about its usefulness?
बिरामीको भूमिका निर्वाह गरेर गर्नुभएको अभ्यास प्रति तपाइको अनुभब कस्तो रह्यो ? तपाइलाई यो कत्तिको उपयोगी लाग्यो ?

2. Do you ever have conversation among colleagues about whether all patients are being treated the same or not?
के तपाई आफ्नो सहकर्मीसँग बिरामीले पाउने समान व्यवहारबारे छलफल गर्नुहुन्छ ?

3. Are there any ways where they get feedback from their patient about how their experience was?
तपाइले दिनुभएको सेवाप्रति बिरामीको कस्तो प्रतिक्रिया पाउनु भएको छ?

4. Do you have anything to add on this other than what we have discussed?

हामीले छलफल गरेको विषयहरूमा तपाईँलाई केही थप कुरा भन्न मन मन लागेको छ ?
