# Supplementary material for: Gender and caste inequalities in primary healthcare usage by under-5 children in rural Nepal: an iterative qualitative study into provider perspectives and the potential role of implicit bias
Source: BMJ Open. 2023 Jun 26;13(6):e069060. doi: 10.1136/bmjopen-2022-069060 (PMC10410982; doi:10.1136/bmjopen-2022-069060)
Supplement: Author's manuscript [file bmjopen-2022-069060.draft_revisions.pdf]

BMJ Open

BMJ Open is committed to open peer review. As part of this commitment we make the peer review history of every article we publish publicly available.

When an article is published we post the peer reviewers' comments and the authors' responses online. We also post the versions of the paper that were used during peer review. These are the versions that the peer review comments apply to.

The versions of the paper that follow are the versions that were submitted during the peer review process. They are not the versions of record or the final published versions. They should not be cited or distributed as the published version of this manuscript.

BMJ Open is an open access journal and the full, final, typeset and author-corrected version of record of the manuscript is available on our site with no access controls, subscription charges or pay-per-view fees (http://bmjopen.bmj.com).

If you have any questions on BMJ Open's open peer review process please email info.bmjopen@bmj.com

BMJ Open

Gender and caste inequalities in primary healthcare utilisation by under-5 children in rural Nepal: Provider perspectives on socio-economic determinants, patient experience, and the potential role of implicit/unconscious bias

Journal:	*BMJ Open*
Manuscript ID	bmjopen-2022-069060
Article Type:	Original research
Date Submitted by the Author:	10-Oct-2022
Complete List of Authors:	Joshi, Saugat ; PHASE Nepal<br>Karki, Alisha; PHASE Nepal<br>Rushton, Simon; The University of Sheffield, Department of Politics and International Relations<br>Koirala, Bikash; PHASE Nepal<br>Basnet, Srijana; PHASE Nepal<br>Rijal, Barsha; PHASE Nepal<br>Karki, Jiban; Liverpool School of Tropical Medicine<br>Pohl, Gerda; PHASE Nepal<br>Baidya, Manish; PHASE Nepal<br>Chater, Tim; University of Liverpool<br>Green, Dan; Aston University<br>Lee, Andrew; The University of Sheffield, School of Health and Related Research
Keywords:	Health policy < HEALTH SERVICES ADMINISTRATION & MANAGEMENT, Quality in health care < HEALTH SERVICES ADMINISTRATION & MANAGEMENT, International health services < HEALTH SERVICES ADMINISTRATION & MANAGEMENT

SCHOLARONE™
Manuscripts

I, the Submitting Author has the right to grant and does grant on behalf of all authors of the Work (as defined in the below author licence), an exclusive licence and/or a non-exclusive licence for contributions from authors who are: i) UK Crown employees; ii) where BMJ has agreed a CC-BY licence shall apply, and/or iii) in accordance with the terms applicable for US Federal Government officers or employees acting as part of their official duties; on a worldwide, perpetual, irrevocable, royalty-free basis to BMJ Publishing Group Ltd ("BMJ") its licensees and where the relevant Journal is co-owned by BMJ to the co-owners of the Journal, to publish the Work in this journal and any other BMJ products and to exploit all rights, as set out in our licence.

The Submitting Author accepts and understands that any supply made under these terms is made by BMJ to the Submitting Author unless you are acting as an employee on behalf of your employer or a postgraduate student of an affiliated institution which is paying any applicable article publishing charge ("APC") for Open Access articles. Where the Submitting Author wishes to make the Work available on an Open Access basis (and intends to pay the relevant APC), the terms of reuse of such Open Access shall be governed by a Creative Commons licence – details of these licences and which Creative Commons licence will apply to this Work are set out in our licence referred to above.

Other than as permitted in any relevant BMJ Author's Self Archiving Policies, I confirm this Work has not been accepted for publication elsewhere, is not being considered for publication elsewhere and does not duplicate material already published. I confirm all authors consent to publication of this Work and authorise the granting of this licence.

For peer review only

Gender and caste inequalities in primary healthcare utilisation by under-5 children in rural Nepal: Provider perspectives on socio-economic determinants, patient experience, and the potential role of implicit/unconscious bias

Corresponding author:

Professor Simon Rushton,

Department of Politics & International Relations, University of Sheffield, Elmfield, Northumberland Road, Sheffield S10 2TU, UK.

Email: simon.rushton@sheffield.ac.uk

Tel: 0114 2221710

Full author list:

Saugat Joshi, PHASE Nepal, Kathmandu, Nepal.

Alisha Karki, PHASE Nepal, Kathmandu, Nepal.

Simon Rushton*, Department of Politics & International Relations, University of Sheffield, Sheffield, UK.

Bikash Koirala, PHASE Nepal, Kathmandu, Nepal.

Srijana Basnet, PHASE Nepal, Kathmandu, Nepal.

Barsha Rijal, PHASE Nepal, Kathmandu, Nepal.

Jiban Karki, Liverpool School of Tropical Medicine, Liverpool, UK.

Gerda Pohl, PHASE Nepal, Kathmandu, Nepal.

Manish Baidya, PHASE Nepal, Kathmandu, Nepal.

Tim Chater, Liverpool Clinical Trials Centre, University of Liverpool, Liverpool, UK.

Dan J Green, Department of Optometry, Aston University, Birmingham, UK.

Andrew Lee, School of Health & Related Research, University of Sheffield, Sheffield, UK.

Keywords: Child Health, Health Services Research, Nepal

Word Count: 4823

For peer review only - http://bmjopen.bmj.com/site/about/guidelines.xhtml

Gender and caste inequalities in primary healthcare utilisation by under-5 children in rural Nepal: Provider perspectives on socio-economic determinants, patient experience, and the potential role of implicit/unconscious bias

Abstract

Introduction: Despite efforts to address health inequalities in Nepal, there are continuing disparities in service utilisation in rural areas by caste and gender. Disadvantaged groups tend to bring their children to the health post for treatment at a later stage, or do not seek healthcare at all. This may be due to various socio-economic determinants such as economic status, level of education, and cultural norms. However, previous negative patient experiences of healthcare can also be influential. Implicit/unconscious bias amongst providers could contribute to such negative experiences. This study explored provider perspectives on: i) why inequalities in service utilisation persist; and ii) their knowledge and understanding of the role of patient experience and implicit/unconscious bias.

Methods: A three stage, iterative qualitative study was conducted involving two rounds of in-depth interviews and a training session with healthcare staff from 22 rural health posts in Nepal. Interview transcripts were analysed using a reflexive thematic approach in relation to the study's aims.

Results: Healthcare providers had high levels of understanding of the cultural, educational and socio-economic factors behind inequalities in healthcare utilisation in their communities.

For peer review only - http://bmjopen.bmj.com/site/about/guidelines.xhtml

However, there was less knowledge and understanding of the role of patient experience - and no recognition at all of the concept of implicit/unconscious bias.

Conclusion: It is highly likely that implicit/unconscious bias affects provider behaviours in Nepal just as it does in other countries. However, there is currently not a culture of thinking about the patient experience and how that might impact on future utilisation of health services. Implicit/unconscious bias training for health students and workers would help create greater awareness of the unintended discriminatory behaviours. This in turn may play a part in improving patient experience, and future healthcare utilisation, particularly amongst disadvantaged groups.

For peer review only - http://bmjopen.bmj.com/site/about/guidelines.xhtml

What is already known on this topic?

- Previous research in Nepal has identified a range of factors that inhibit health service utilisation amongst certain groups including parental education, preference for traditional healers, distance to health centre, differential treatment of male and female children, and patent experience (with a poor experience being associated with a reluctance to return in future).
- Evidence from other countries identifies implicit/unconscious bias amongst healthcare professionals as being detrimental to patient experience.

What this study adds?

- Existing research on health service utilisation in Nepal has been from the patient perspective: this study examines service providers' perspectives on the factors that cause inequalities in health service utilisation.
- Healthcare providers have a good understanding of the social and economic factors that determine service utilisation, and can point to instances of discriminatory treatment.
- Yet they have little knowledge (and have received little training) on the importance of patient experience - including the potential role of implicit/unconscious bias amongst providers themselves.

How this study might affect research practice or policy?

- Research is needed into the extent, consequences and drivers of implicit/unconscious bias in the particular context of Nepal - as well as to explore effective ways to counter this.

For peer review only - http://bmjopen.bmj.com/site/about/guidelines.xhtml

- Implicit/unconscious bias training (in health/medical schools and for government and non-government health staff) would be a first step in creating greater awareness about unintended discriminatory behaviours, improving patient experience as well as future healthcare utilisation amongst disadvantaged groups.

For peer review only

Introduction

The world has made substantial progress in reducing childhood mortality and child survival over the past three decades. Globally, the under-5 mortality rate has decreased by 60%, from 93 deaths per 1000 live births in 1990 to 37 in 2020 [1]. Nevertheless, around 5 million children still die each year, largely from causes that are preventable [1]. The health of young children is especially susceptible to socioeconomic inequalities, which have led to significant differences in childhood morbidity and mortality between and within countries [2].

Among the countries of South Asia, Nepal has the highest reported prevalence of under-5 mortality over the past 15 years [3]. In recognition of this, child health is one of the priority programs of the government, which has targeted reducing under-5 mortality to 28 per 1,000 live births and neonatal mortality to 12 per 1,000 live births by 2030 [4]. As a result of this prioritisation, significant improvements have been made in child health services and outcomes over recent years [5]. Despite this, socio-economic inequalities remain an obstacle to tackling Nepal's continuing high rates of child illness and death [6]. In addition to continuing inequalities in the socio-economic determinants of health, health services are still not effectively distributed to all individuals, most commonly failing to reach those from the low castes, ethnic minorities, people living in remote areas, and other marginalised populations [7]. Deeply-rooted, societal, gender, caste/ethnic, regional and wealth inequities have made it difficult to address this situation.

Health care utilisation by children under-5 depends on the health-seeking behaviours of their parents and caregivers. As seen in studies elsewhere in South Asia, parents and caregivers do not always appropriately seek health care services for childhood illness for a variety of reasons, including low levels of parental education [8, 9, 10], a preference for traditional healers [11],

For peer review only - http://bmjopen.bmj.com/site/about/guidelines.xhtml

and living long distances from a health centre [12]. Health-seeking patterns for children have also been found to be highly gendered in Nepal [13], as in other Asian countries [14, 15]. Delays in care-seeking can lead to illnesses becoming more severe, or in some cases mean that the child does not receive treatment at all.

Stigma is one of the causes of health inequalities [16], and discriminatory attitudes and practices among health service providers can also discourage healthcare utilisation by certain groups. For example, although caste-based discrimination is illegal in Nepal, continuing cultural belief in the 'untouchability' of Dalits has been found to limit access to healthcare services [17]. When patients do present at a health facility, studies in other settings have shown that patients' experience can significantly affect future utilisation: if people are dissatisfied with services, or feel discriminated against when using them, they are less likely to return [18, 19, 20]. One study conducted in Nepal found that only one-third (35.9%) of respondents reported receiving good communication from health service providers [21], suggesting that shortcomings in patient experience are widespread. Ensuring a good patient experience for all is therefore an important element of attempts to promote health service utilisation [22].

However, this is an under-researched area in Nepal. Relatively few studies have examined the barriers to healthcare utilisation for under-5s in Nepal, with those that have examined socio-economic factors affecting the likelihood of people seeking health services, from the perspective of patients [10, 11]. In this study, we seek to complement this previous work by: i) adopting a service provider-focussed perspective, to understand their perceptions on the reasons for patterns of health care utilisation; and ii) to explore service providers' perspectives on patient experience and how this may relate to service utilisation by different groups. This qualitative study builds on the findings of a previous quantitative study in which patient records from 23 rural health posts

For peer review only

For peer review only - http://bmjopen.bmj.com/site/about/guidelines.xhtml

were studied to understand inequalities in health service utilisation, diagnosis and treatment for under-5 children [23]. The data revealed significant differences by gender and caste but did not explain *why* those differences occurred. In the follow-up study reported here, we conducted in-depth interviews (IDIs) with service providers from those same health posts to explore their perceptions as to why these differences exist in their communities, and subsequently designed and implemented training sessions based on role-play scenarios to increase their knowledge and understanding of patient experience.

Methods

Study design and setting

We applied a qualitative approach in this study, employing IDIs with health workers from rural health posts to explore the reasons for inequalities in service utilisation, focusing on providers' perceptions of the issues within the rural communities in which they work. After the initial round of analysis identified notable gaps in the data around the potential effect of service providers' own attitudes and practices, we designed and implemented training and subsequently conducted a second round of IDIs to explore interviewees' awareness and understanding of unconscious bias.

The research participants were clinical staff members drawn from 22 of the 23 rural health posts that had been part of the preceding quantitative study (for logistical reasons, it was not possible to interview staff from one health post). We report this study in accordance with the consolidated criteria for reporting qualitative research (COREQ).

For peer review only - http://bmjopen.bmj.com/site/about/guidelines.xhtml

Data collection method and Research Participants

We initially conducted In-Depth Interviews (IDIs) with one health service provider currently working in each of 22 health posts spread across four provinces of Nepal (Bagmati, Gandaki, Karnali and Sudurpaschim provinces), covering the rural hill districts of Gorkha, Sindhupalchok, Mugu, Humla and Bajura. These particular health posts were selected as they are currently supported by the NGO partner organisation involved in this study. They are staffed by a mixture of government staff and staff of the NGO partner (name withheld for anonymisation). All of the interviewees involved in this study were members of NGO staff. Interviewees included Auxiliary Nursing Midwives (ANMs), Staff Nurses and Paramedics. The majority had experience of having worked in multiple rural health posts. The characteristics of the interviewees are presented in Table 1.

Table 1: Characteristics of interviewees

Participant code	Age	District	Sex	Ethnicity	Education qualification	Overall experience (years)
KII_F_01	26	Gorkha	F	Janajati	Intermediate	4
KII_F_02	35	Mugu	F	Brahmin/Chhetri	Staff Nurse	5
KII_F_03	27	Sindhupalchok	F	Brahmin/Chhetri	Intermediate	5.5
KII_F_04	27	Mugu	F	Brahmin/Chhetri	ANM	4
KII_F_05	26	Bajura	F	Brahmin/Chettri	ANM	6

For peer review only - http://bmjopen.bmj.com/site/about/guidelines.xhtml

KII_F_06	26	Mugu	F	Dalit	ANM	4
KII_M_07	25	Mugu	F	Brahmin/Chhetri	ANM	4
KII_F_08	25	Humla	F	Dalit	ANM	8
KII_F_09	30	Bajura	F	Brahmin/Chhetri	Staff Nurse	10
KII_M_10	34	Mugu	M	Brahmin/Chhetri	HA	14
KII_F_11	26	Mugu	M	Dalit	HA	5
KII_F_12	28	Mugu	F	Brahmin/Chhetri	ANM	7
KII_F_13	33	Mugu	F	Brahmin/Chhetri	ANM	7
KII_F_14	32	Mugu	F	Janajati	ANM	7
KII_F_15	24	Mugu	F	Brahmin/Chhetri	ANM	2
KII_F_16	30	Humla	F	Janajati	Staff Nurse	10
KII_F_17	31	Sindhupalchok	F	Janajati	ANM/Intermediate pass	8
KII_M_18	28	Mugu	M	Brahmin/Chhetri	Health Assistant	8
KII_F_19	28	Bajura	F	Dalit	ANM	10
KII_F_20	29	Bajura	F	Brahmin/Chhetri	ANM/10+2	10
KII_F_21	28	Humla	F	Brahmin/Chhetri	ANM/ 10+2	10
KII_F_22	32	Bajura	F	Janajati	ANM	13

Data collection

Data collection was via a three-stage iterative process in which the research plans were adapted in response to initial findings.

The initial round of interviews was undertaken during a week of training events attended by all health staff of the project's NGO partner (which was why only NGO staff, and not government staff, participated). Prior to the IDIs, the results of the preceding quantitative study (i.e. the data

For peer review only - http://bmjopen.bmj.com/site/about/guidelines.xhtml

on gender, geographic and caste inequalities in these health posts) were presented to the health staff. They were then interviewed individually, according to the pre-tested schedule, to ascertain their perspectives on *why* these inequalities persist.

The semi-structured interview schedule was developed in English and then translated into Nepali. We conducted four interviews as a pre-test and made necessary changes to the interview schedule to improve its clarity. We then conducted the 18 remaining IDIs. In a small number of cases (where IDIs were not possible during the training event for practical reasons), the interviews were conducted later via telephone.

A rapid analysis of the emerging findings from this first round of 22 interviews was discussed by the members of the research team, and omissions/notable gaps in the data were considered [24], taking into account the published evidence on health service utilisation (in Nepal and elsewhere). The potential role of negative patient experience and unconscious bias leading to suboptimal patient experience – even where providers themselves had the best of intentions - was identified as a notable silence in the data.

In the second stage, in response to these emerging findings, a training session on patient experience was designed and implemented. This included a role play session conducted in pairs in which half of the participants were asked to play the role of a patient bringing a baby to a health post, and the other half to play the role of the service provider. After the consultation was complete, the participants swapped roles. This was followed by a guided de-brief plenary discussion in which patient experience was discussed, and participants' perspectives on necessary elements of a positive patient experience were collated. This session was designed to prompt reflection amongst participants on patient experience.

For peer review only - http://bmjopen.bmj.com/site/about/guidelines.xhtml

In the third stage (after the training event had finished and participants had returned to their health posts), a new interview guide was developed focusing on the themes of patient experience and participants' knowledge and understanding of implicit/unconscious bias. Based on this guide, a second round of follow-up interviews was conducted, via telephone, with all 22 study participants.

All interviews took place in April and May 2022, carried out by five experienced qualitative researchers (AK, SJ, BR, SB and BK). The training session additionally involved facilitation by JK, GP and SR. We obtained written consent from participants. Both the face-to-face interviews and the telephone interviews were audio recorded with the participants' consent. The average length of the interviews was 25 minutes.

We first transcribed the recording verbatim into Nepali, and then translated these transcriptions into English. All personal identifiers were replaced with unique codes (see Table 1). Seven of the interview transcripts were back translated to ensure that no meaning was lost during the translation process.

Data analysis

Two researchers (AK and SJ) coded the interview transcripts using a reflexive thematic approach in NVivo 12 (Version 12 pro, QRS International) qualitative data management software [25]. The researchers read the full transcripts before starting the coding process. Following this, we organised all the text from the interviews by coding and grouping them into appropriate themes, supported by discussion amongst the members of the research team. During the process, we developed initial sets of codes while thoroughly reading the text followed by combining and

For peer review only - http://bmjopen.bmj.com/site/about/guidelines.xhtml

modifying them, thus developing a more refined codebook. Finally, we grouped the codes into major thematic categories and the whole research team finalised the codebook for the data analysis process.

Ethical considerations

Ethical approval for the study was obtained from (Anonymised for peer review) and the Ethical Review Board of the Nepal Health Research Council (NHRC). See the supplementary file 'Reflexivity Statement' for further reflections on the wider ethical aspects of the project.

Patient and Public Involvement

This research focuses on the perceptions of health workers, who consented to their participation in the study. It was not appropriate or possible to involve patients or the public in the design, or conduct, or reporting, or dissemination plans of our research

Findings

First round of interviews: Provider explanations for differential utilisation of health services

In this section, we present the explanations given by service providers in the first round of interviews for the observed differences in health service utilisation in the areas in which they work according to i) caste and ii) gender.

For peer review only

i) Caste

Lack of knowledge and education

Interviewees indicated that, in their experience, Dalits in particular lacked awareness, knowledge and education on the causes, symptoms, and treatments for diseases.

> *"When a brahmin child gets sick, mostly, the parents bring them to the health centre as soon as possible. But people belonging to the Dalit caste are not educated nor aware enough to make such informed decisions.* KII_F_08

> *"People of lower caste are uneducated and are unaware of their health. Janajati people make the children drink local alcohol to treat their condition".* KII_F_22

Economic factors

The interviewees noted that, in comparison to people from other castes, Dalits tend to be poorer.

> *The economic situation of Dalits is also poor compared to other groups, which is why they tend to seek household remedies to cure the illnesses of their children".* KII_F_08

Although services are provided free of charge, interviewees explained that economic deprivation could nevertheless affect service utilisation: those from lower castes have less flexibility with their time, often having to work long hours in their own fields and/or for other people to make a

For peer review only - http://bmjopen.bmj.com/site/about/guidelines.xhtml

living. As a result, they are not able to visit the health post without incurring financial losses, and thus tend not to bring their children for treatment in the early stage of illness.

Preferential use of traditional healers

Interviewees reported that traditional healers' services are often utilised at the community level, either because they are preferred by patients, or because they are more conveniently located.

> *"In rural areas, faith healers are still the first choice for the majority of people. People who live near health posts try to bring their children as soon as possible, but people who live far from the village centre first try traditional remedies and consume medicinal herbs".* KII_F_08

> *"People who live far from the health post prefer to take their children to a faith healer to treat their condition. They only come to a health facility if they are referred by the faith healer, or if the faith healer is not able to treat their condition".* KII_F_06

Interviewees reported that older people and those who are less educated (often from lower castes) are particularly likely to believe in gods and goddesses and prefer to participate in rituals and make offerings in case of illness. These groups were seen as more likely to take their children to traditional healers, and health education efforts were seen as having had little impact so far:

> *"We are involved in conducting household programs to raise the awareness level and educate people about the importance of their children's health. We ask them to bring*

For peer review only - http://bmjopen.bmj.com/site/about/guidelines.xhtml

their children [to the health post] as soon as they get sick. However, people in the village are still following outdated practices that were taught by their ancestors." KII_F_10

Deep rooted (and internalised) stigma

The deep-rooted stigma and discrimination against Dalits in the community was seen as reducing their willingness to bring their children to the health post, resulting in self-exclusion from services. When they do bring their children to the health post, interviewees reported that other patients (for example, those from the Brahmin/Chhetri caste) insist that their children should be treated first. Interviewees reported that many Dalits themselves believe that it is a sin if they touch an upper caste person, so even when they do go to the health post, they sit in a corner, away from other waiting patients. One of our participants said that:

"When we demonstrated how 'jaulo' [a kind of porridge] is made, we invited all the people from different castes. When they came, all the so-called upper caste people sat on one side and the lower-class people sat on the other side. While eating food, they did not touch each other. It's not like people set a rule about such things, but the culture was such that a Dalit person believes that it is not nice to touch a person of upper caste". KII_F_06

ii) Gender

Differential attitudes towards male and female children

For peer review only - http://bmjopen.bmj.com/site/about/guidelines.xhtml

Providers believed that parents are more likely to take sons to health facilities quickly when they are sick, and spend more money on their care than they do for their daughters. They reported that parents of daughters are less likely to seek care outside of home and, even when seeking care, they are more likely to go to an informal or traditional healer. Even mothers were seen as being more vigilant about the health of their sons.

> *"There was a woman in Bajura district who had three daughters. One of her daughters had severe pneumonia, but she did not bring her to the health post. She said that it would not matter if one daughter dies because she was still of childbearing age and could give birth to another one if this one dies"* KII_F_03

> *"In the community I worked in, there was a woman who had a son after four daughters. She didn't bring any of her daughters when they got ill. But she used to get worried and concerned and brought her son if he suffered even from a minor cold. She used to say that after waiting and praying for a long time, they were able to have a son. She brought her son as if it was a very serious case, but it was normal"* KII_F_02

Service providers attributed these differences in health-seeking behaviours to deeply-rooted cultural norms around the relative value of male and female children, and their expected future roles and contribution to the family.

> *"A girl child doesn't even get a naming ceremony. A mother gives a random name based on what she likes"*. KII_F_08

> *"People say that a daughter is not going to earn and she will not achieve anything big in life, so they tend to deprive a female child of education as well as not seeking health*

For peer review only

For peer review only - http://bmjopen.bmj.com/site/about/guidelines.xhtml

services. They say that there is no point in educating a daughter since she will get married after a certain age." KII_F_06

"People believe that a male child can carry the legacy of their parents forward. Sometimes, when we are not able to treat their daughters, we refer them to a hospital. When we do so, they ask us if we can treat her or not. When we say no, then they don't think it is worth taking her to the hospital. Even if the daughter dies, the mother does not feel guilty." KII_F_06

Second round of interviews: Interviewees' knowledge and understanding of patient experience and implicit/unconscious bias

As described above, the first round of interviews revealed that interviewees had a very good awareness of the ways in which caste and gender could affect health service utilisation in the communities in which they were working. They did not, however, identify the potential role of provider-side attitudes and behaviours. The training session on patient experience and the follow-up interviews explored these themes in depth. In the second round of interviews, we examined interviewees' experience of discriminatory behaviours, and their knowledge and understanding of the potential impact of uncon scious bias on their own treatment of patients.

Experiences of unequal patient experience

For peer review only - http://bmjopen.bmj.com/site/about/guidelines.xhtml

When asked about the respective experiences that different groups may have when visiting a health post, many interviewees were able to give examples of having witnessed colleagues not treating every patient equally.

> *"It is not so common but, in some cases, health personnel tell a Dalit patient to keep their distance and ask them to do things on their own, just to avoid touching them as they are considered untouchables."* KII_F_08

> *"When I was working in a remote area of Gorkha district, Dalit children were not allowed to sit with other patients and they received their check-up only after Brahmin and Chhetri children had been seen"*. KII_F_16

> *"People of certain castes were not given the respect they deserve and staff [government health workers] mostly tried to ignore them as much as possible, just to avoid touching them. When we [NGO staff] tried to touch them to treat them, we were told to not to touch such patients and to avoid physical contact"* KII_F_09

> *"As most of the [government] staff working in the health facility are local, they treat patients based on their caste/ethnicity. People who are from the upper castes are treated with utmost care and respect and those who belong to lower castes are neglected and, sometimes, mistreated as well"*. KII_F_09

In all cases, such discriminatory treatment was attributed to others - and in all cases to government health staff rather than staff of the NGO (see Limitations).

Although, when asked in the training session and interviews, participants agreed with the logic that patient experience could have an impact on whether or not patients chose to return to the health post in case of future illness amongst their children, most indicated that there was no

For peer review only - http://bmjopen.bmj.com/site/about/guidelines.xhtml

effective feedback mechanism in the health facilities where they work to capture patients' (dis)satisfaction with their experience.

> *"We know that from their body language. We don't have any specific mechanism, but whenever a patient tells us then we get to know about their experiences".* KII_F_02

Furthermore, interviewees said that they had not previously received training relating to patient experience. Reflecting on a role play-based training session provided to participants by the research team, one reflected that

> *"Previously, we used to see things from the side of a health service provider, but this roleplay session made us realise that we need to treat patients with patience. We do not have much patience as we have to see many patients in a day. I think [this training] will definitely support us in our work in the future."* KII_F_05

Knowledge and understanding of implicit/unconscious bias

Although discriminatory treatment was recognised (and always attributed to others), interviewees reported that they had never heard about the concepts of unconscious or implicit/unconscious bias, nor received any training on it. For example,

> *"I don't think I have heard about such a term. We don't intentionally try to be biased towards a particular individual. However, there might be some cases that we are not aware of" KII_F_09*

> *"I don't think I have heard or had such an experience about implicit bias in my professional life" KII_F_07*

For peer review only

"I have not received any specific training on that. In other training that we received, we are only taught about treating the patient right but we are not aware of the bias related training that you mentioned."KII_F_04

Discussion

We found that rural healthcare providers had a high level of awareness and understanding of the cultural, educational, and socio-economic factors behind inequalities in healthcare utilisation. The lack of awareness, knowledge and education amongst the Dalits and other low castes, their preference for traditional healers, deep-rooted and internalised stigma, and differential parental attitudes towards male and female children, were all commonly identified, and were in accordance with findings in the existing literature from Nepal [6, 7, 10, 11, 13, 26, 27] and elsewhere in South Asia [8, 9, 12, 15-17, 28].

However, participants tended to have far low levels of awareness, understanding and training on the ways in which patient experience might adversely impact service utilisation. This is despite the fact that many respondents reported witnessing discriminatory behaviour by other health service providers - they generally believed that they themselves would treat everyone equally in their own practice (see 'Limitations' for our reflections on self-serving bias here). Studies from other countries have demonstrated that poor (and especially discriminatory) patient experience can deter future health service utilization, as well as affect treatment adherence [18-20, 22, 29-31]. However, our findings suggest that in Nepal there is not a culture of thinking about the patient experience.

For peer review only - http://bmjopen.bmj.com/site/about/guidelines.xhtml

Most previous studies on the role of implicit/unconscious bias have taken place in the Global North. For example, a narrative review indicated that healthcare providers in the United States show implicit/unconscious negative attitudes and stereotypes about several stigmatised groups [32]. Another study reported that the majority of health professionals showed preferential treatment towards non-Hispanic white children or adults over American Indian patients [33]. These studies suggest that being a child does not protect one from being on the receiving end of unconscious bias. Although we did not utilise methods that could have demonstrated implicit/unconscious bias amongst our participants, the existing literature from elsewhere suggests that it is extremely unlikely that our interviewees are completely free from such unconscious bias. This leads us to suspect that bias is currently unrecognised rather than absent.

The fact that unconscious bias is frequently unrecognised by those who hold such biases is well known. Numerous studies have shown that self-reporting of unconscious bias is not reliable as it is challenging for individuals to critically evaluate their own thoughts [34, 35]. Such biases exist in everyone, even well-intentioned healthcare workers (and also, of course, the members of this research team). Evidence indicates that healthcare professionals have the same levels of implicit/unconscious bias as the general public [36]. Healthcare providers' awareness of implicit/unconscious bias is not well documented in the context of Nepal and our literature review revealed very few previous studies in Nepal gave this topic even a passing mention [37; for an exception see 38]. However, it is evident that unconscious bias is significantly related to patient-provider interactions, treatment decisions, and patient health outcomes in all contexts [33], and certainly will be in Nepal.

This study has revealed a need for further research on implicit/unconscious bias in healthcare settings in Nepal. Whilst the literature from other countries suggests that it is highly likely that

For peer review only - http://bmjopen.bmj.com/site/about/guidelines.xhtml

implicit/unconscious bias impacts on patient treatment in Nepal, it is important to understand the extent, consequences and drivers of this bias in the particular context of Nepal - as well as to explore effective ways to counter this.

Our findings also have implications for practice as implicit/unconscious bias has the potential to affect not only treatment outcomes, but also whether patients (especially those from disadvantaged groups) bring their children to health facilities for treatment in future. Studies from other countries have shown that the provision of training to increase the awareness of individuals about their own unconscious biases can have a positive impact, equipping them to better manage and account for these biases in future [39]. Many different approaches to such training are available. To take one example, the Implicit Association Test (IAT), created in 1998, is the most widely used computerised online tool for measuring and bringing awareness of unconscious bias [40]. Neither the IAT nor any of the myriad other forms of training and awareness-raising about implicit/unconscious bias have, to our knowledge, been widely utilised in Nepal – including amongst healthcare workers. Although training cannot by itself completely remove bias, confronting health service providers with evidence of their unconscious biases and providing training on how to counter these biases is a necessary first step [32, 40]. We would recommend that Nepalese health/medical schools, the government of Nepal and other employers of health service providers in the country investigate the possibility of introducing implicit/unconscious bias training.

Limitations

For peer review only - http://bmjopen.bmj.com/site/about/guidelines.xhtml

For peer review only

We acknowledged that there are limitations with our study. This study drew on the perspectives of 22 healthcare providers from selected rural health posts in Nepal. These findings do not necessarily reflect the perspectives of all healthcare providers in all areas of the country. Furthermore, due to the nature of the study participants included only staff employed by an NGO that supports these health posts, and not the government health staff that they work alongside. It was notable that participants did not provide examples of discriminatory behaviours by themselves or their NGO colleagues: such behaviours were always attributed either to members of the community or to government health staff. Although our methods do not allow us to demonstrate that this is false, there is a risk of self-serving bias here, and this aspect of the results should be handled with care.

This study did not examine the experiences of patients, parents or caregivers who receive services: this would be an important avenue for future research.

Finally, this study only involved staff from Primary Healthcare Centers (health posts), and may not be generalizable to other healthcare settings such as hospitals.

Conclusion

The impact that provider-side factors may have on health service utilisation in Nepal has not been widely studied, and this is, to our knowledge, the first paper to explore service providers' perspectives on the potential roles of patient experience and unconscious bias in deterring health seeking for young children.

In some ways, the study make uncomfortable reading for the NGO that participated in this study. However, we would argue that there is a more positive way of interpreting these findings. Given

For peer review only - http://bmjopen.bmj.com/site/about/guidelines.xhtml

what is known about the difficulties of relying on self-reported biases, implicit/unconscious bias is likely to be an issue across all health workers in Nepal and not specific to this particular NGO. This NGO is the first in Nepal, to our knowledge, to properly investigate this issue. Following on from the training provided in patient experience, the next step in this process - in line with our recommendation above - would be for the NGO to pursue the wider use of implicit/unconscious bias training with staff, in order to provide them with the practical awareness and skills to recognize and mitigate their own biases as well as their unintentional involvement in the perpetuation of discrimination.

Competing interests statement

Authors SJ, AK, BK, SB, BR, JK, GP and MB are either currently or previously employed by PHASE Nepal, the NGO partner for this study and the employer of the study participants.

Funding statement

This research was supported by the UK Economic and Social Research Council's GCRF NGO Secondary Data Analysis Initiative, Grant Ref: ES/T010436/1.

Data Availability statement

No additional data available

Contribution Statement

SR, JK, GP, MB, TC, DJG and AL all contributed to the planning of the wider project of which this is part.

For peer review only

For peer review only - http://bmjopen.bmj.com/site/about/guidelines.xhtml

SJ, AK, SR, BK, CB, BR, JK and GP contributed to the planning and design of the qualitative component of the study which is reported in this manuscript.

SJ, AK, BK, SB and BR conducted the fieldwork.

SJ, AK, SR and JK undertook the initial analysis after the first round of interviews.

AK, SJ, SB, BR, BK, SR, JK and GP conducted the subsequent analysis.

SJ and AK, with support from SR, BK and JK, led on writing the first draft of the manuscript.

All authors contributed to the final version of the manuscript.

SR submitted the study and has overall responsibility for the overall content.

References:

1. WHO. *Child mortality (under 5 years)*. 2022 Available from: https://www.who.int/news-room/fact-sheets/detail/levels-and-trends-in-child-under-5-mortality-in-2020. Accessed 12 September 2022

2. Barros, FC, Victora CG, Scherpbier R, Gwatkin D. Socioeconomic inequities in the health and nutrition of children in low/middle income countries. *Revista de Saúde Pública* 2010; 44 (1). https://doi.org/10.1590/S0034-89102010000100001

3. Sohail H, Neupane S. Prevalence of and factors associated with under-5 mortality in South Asia. *International Health* 2018;11(2):119-127.

For peer review only - http://bmjopen.bmj.com/site/about/guidelines.xhtml

4. National Planning Commission (Nepal). *Nepal's Sustainable Development Goals, Baseline Report, 2017.* Kathmandu, Nepal: Government of Nepal/National Planning Commission, 2017.

5. Ministry of Health. *Nepal's Every Newborn Action Plan (2016)*. Kathmandu, Nepal: Ministry of Health, 2016. https://www.healthynewbornnetwork.org/hnn-content/uploads/NENAP-final-lowresolution.pdf.

6. Pandey JP, Dhakal MR, Karki S, Poudel P, Pradhan MS. *Maternal and Child Health in Nepal: The Effects of Caste, Ethnicity, and Regional Identity: Further analysis of the 2011 Nepal Demographic and Health Survey, 2013*. Calverton, Maryland, USA: Nepal Ministry of Health and Population, New ERA, and ICF International: 2013. https://www.dhsprogram.com/pubs/pdf/FA73/FA73.pdf. Accessed 12 September 2022.

7. Ghimire U, Manandhar J, Gautam A, Tuladhar S, Prasai Y, Gebreselassie T. *Inequalities in Health Outcomes and Access to Services by Caste/Ethnicity, Province, and Wealth Quintile in Nepal.* DHS Further Analysis Reports No. 117. Rockville, Maryland, USA: ICF: 2019.

8. Kachoria AG et al. The association of religion with maternal and child health outcomes in South Asian countries. *PLoS One* 2022; 17(7): e0271165.

9. Khanam M, Hasan E. Inequalities in health care utilization for common illnesses among under five children in Bangladesh. *BMC Pediatrics* 2020; 20(1): 192.

10. Sreeramareddy CT, Shankar RP, Sreekumaran BV et al. Care seeking behaviour for childhood illness- a questionnaire survey in western Nepal. *BMC Int Health Hum Rights* 2006;6, 7. https://doi.org/10.1186/1472-698X-6-7

11. Pokhrel S, Sauerborn R. Household decision-making on child health care in developing countries: the case of Nepal. *Health Policy and Planning* 2004;19(4):218–233.

For peer review only - http://bmjopen.bmj.com/site/about/guidelines.xhtml

12. Sreeramareddy CT., Sathyanarayana T, Kumar HH. Utilization of health care services for childhood morbidity and associated factors in India: a national cross-sectional household survey. *PLoS One* 2012; 7(12): e51904.

13. Pokhrel S et al. Gender role and child health care utilization in Nepal. *Health Policy* 2005; 74(1):100-9.

14. Yan X, Ren Q. Selective neglect: Gender disparities in children's healthcare utilization in rural China. *Chinese Journal of Sociology* 2019; 5(3): 283-311.

15. Ismail SA, McCullough A, Guo S, et al. Gender-related differences in care-seeking behaviour for newborns: a systematic review of the evidence in South Asia. *BMJ Global Health* 2019;4:e001309.

16. Thapa R, van Teijlingen E, Regmi PR, Heaslip V. Caste Exclusion and Health Discrimination in South Asia: A Systematic Review. *Asia Pacific Journal of Public Health* 2021;33(8):828-838.

17. Thapa R., et al., Caste exclusion and health discrimination in South Asia: A systematic review. *Asia Pacific Journal of Public Health* 2021; 33(8): 828-838.

18. Chapman EN, Kaatz A, Carnes M. Physicians and implicit bias: how doctors may unwittingly perpetuate health care disparities. *Journal of General Internal Medicine* 2013; 28(11):1504-1510.

19. Narayan MC. Addressing Implicit Bias in Nursing: A Review. *American Journal of Nursing* 2019; 119(7).

20. Kelley JM et al. The influence of the patient-clinician relationship on healthcare outcomes: a systematic review and meta-analysis of randomized controlled trials. *PLoS One* 2014; 9(4): e94207.

For peer review only - http://bmjopen.bmj.com/site/about/guidelines.xhtml

21. Trimmer C, Målqvist M. Clinical communication and caregivers' satisfaction with child healthcare in Nepal; results from Nepal Health Facility Survey 2015. *BMC Health Services Research* 2019; 19(1):1-8.

22. Baidya M, Gopichandran V, Kosalram K. Patient-physician trust among adults of rural Tamil Nadu: a community-based survey. *Journal of Postgraduate Medicine* 2014; 60(1): 21.

23. REFERENCE REMOVED FOR ANONYMISATION

24. Poland B, Pederson A. Reading Between the Lines: Interpreting Silences in Qualitative Research. *Qualitative Enquiry* 1998; 4(2): 293-312

25. Hilal AH, Alabri SS. Using NVivo for data analysis in qualitative research. *International Interdisciplinary Journal of Education* 2013; 2(2): 181-186.

26. French AN. Dalits and mental health: Investigating perceptions, stigma and barriers to support in Kathmandu, Nepal. *Journal of Global Health Reports* 2020;4:e2020009.

27. Nepali KB. Untouchability and Caste Based Discrimination in Nepal. *Nepalese Journal of Participatory Development* 2018; 20(18): 49.

28. Barcellos SH, Carvalho LS, Lleras-Muney A. Child gender and parental investments in India: Are boys and girls treated differently? *American Economic Journal: Applied Economics* 2014;6(1):157-89.

29. Hausmann LR et al. Perceived discrimination in health care and health status in a racially diverse sample. *Medical Care* 2008.;46(9): 905.

30. Lee C, Ayers SL, Kronenfeld JJ. The association between perceived provider discrimination, health care utilization, and health status in racial and ethnic minorities. *Ethnicity & Disease* 2009; 19(3): 330.

For peer review only - http://bmjopen.bmj.com/site/about/guidelines.xhtml

31. Burgess DJ et al. The association between perceived discrimination and underutilization of needed medical and mental health care in a multi-ethnic community sample. *Journal of Health Care for the Poor and Underserved* 2008l 19(3): 894-911.

32. Zestcott CA, Blair IV, Stone J. Examining the Presence, Consequences, and Reduction of Implicit Bias in Health Care: A Narrative Review. *Group Processes & Intergroup Relations* 2016; 19(4): 528-542.

33. Hall WJ, Chapman MV, Lee KM, Merino YM, Thomas TW, Payne BK, Eng E, Day SH, Coyne-Beasley T. Implicit racial/ethnic bias among health care professionals and its influence on health care outcomes: a systematic review. *American Journal of Public Health* 2015;105(12):e60-76.

34 Greenwald AG, Banaji MR. Implicit social cognition: attitudes, self-esteem, and stereotypes. *Psychological Review* 1995;102(1): 4.

35. Dang J, King KM, Inzlicht M. Why Are Self-Report and Behavioral Measures Weakly Correlated? *Trends in Cognitive Sciences* 2020; 24(4):267-269.

36. FitzGerald C, Hurst S. Implicit bias in healthcare professionals: a systematic review. *BMC Medical Ethics* 2017; 18(1):1-18.

37. Ashish KC, Moinuddin M, Kinney M, Sacks E, Gurung R, Sunny AK, et al. Mistreatment of newborns after childbirth in health facilities in Nepal: Results from a prospective cohort observational study. *PLoS One* 2021; 16(2): e0246352.

38. Tergesen CL, Gurung D, Dhungana S, Risal A, Basel P, Tamrakar D, Amatya A, Park LP, Kohrt BA. Impact of Service User Video Presentations on Explicit and Implicit Stigma toward Mental Illness among Medical Students in Nepal: A Randomized Controlled Trial. *International Journal of Environmental Research and Public Health* 2021; 18(4):2143.

For peer review only - http://bmjopen.bmj.com/site/about/guidelines.xhtml

39. Marcelin JR, Siraj DS, Victor R, Kotadia S, Maldonado YA. The impact of unconscious bias in healthcare: how to recognize and mitigate it. *Journal of Infectious Diseases* 2019;220(Supplement 2):S62-73.

40. Arif SA, Schlotfeldt J. Gaps in Measuring and Mitigating Implicit Bias in Healthcare. *Frontiers in Pharmacology* 2021;12: 633565-633565.

For peer review only

For peer review only - http://bmjopen.bmj.com/site/about/guidelines.xhtml

635

For peer review only

Reflexivity Statement

1. How does this study address local research and policy priorities?

The research project from which this study arises was co-developed by a Nepali NGO (anonymised here for peer review) and researchers from the UK as part of a long-term and ongoing research partnership. The project sought to utilise data held and generated by the NGO to further understand health inequalities in the communities in which it works to support health service delivery, in order to improve future service provision.

The study reported here built upon a previous study, and responded to the Nepali NGO partner's desire to further understand the reasons for inequalities that study found in service utilisation, with the aim of identifying potential actions/improvements to address under-utilisation by some groups.

2. How were local researchers involved in study design?

The original quantitative project was co-developed by staff of the Nepali NGO (MD, JK, GP) and UK-based researchers (SR, TC, DJG, AL, JK). This qualitative follow-on study was co-designed by researchers from both institutions (SJ, AK, BK, SB, BR, GP; SR, JK) in response to analysis of the quantitative data.

Nepali researchers (SJ, AK, BK, SB, BR) led on the design of the interview guides for this study, with support from non-Nepali researchers (SR, GP). SR, JK and GP - all experienced trainers - led on the design of the training session.

Some of the high-income country researchers involved in the research (SR, AL) have long track-records of research in Nepal and other low- and middle-income countries. This has been made possible due to privileged access to research funding, which has allowed for the development of this (and other) international partnerships and the resourcing of research that would not have been possible without them leveraging their privilege. To some extent, the same is true of JK who, although Nepali, has studied and now works in a high-income country academic institution. GP is originally from a high-income country background, but has lived and worked in Nepal for two decades. Nevertheless, her background undoubtedly puts her in a privileged position to develop international partnerships. TC and DJG were working in Nepal for the first time on this project.

High-income country researchers are over-represented on the list of authors of this article, precisely due to our privileged positions – especially as researchers with experience in international research. We are ourselves implicated in the institutions that perpetuate inequities through parachute research, and are therefore committed to addressing the problem.

3. How has funding been used to support the local research team?

The funding of this project directly contributed to the salaries of staff from the partner NGOs working on the project (SJ, AK, BK, SB, BR, MB), helping the NGP partner to retain and develop their research capacity. This has included being supported by the senior authorship team to develop skills and networks, and to be listed as authors on the current manuscript.

4. How are research staff who conducted data collection acknowledged?

For peer review only

All researchers involved in data collection (SJ, AK, BK, SB, BR) are authors of this manuscript.

5. Do all members of the research partnership have access to study data?
All members of the partnership have access to data.

6. How was data used to develop analytical skills within the partnership?
The iterative nature of the research design allowed for collaboration between Nepali and high-income country researchers in the development of the original interview schedules; the analysis of that data; and the subsequent design of the training session and second round of interviews. This analysis was a collective effort, with no distinction being made between Nepali and non-Nepali researchers.

7. How have research partners collaborated in interpreting study data?
This manuscript was planned during a face-to-face meeting following initial analysis of the data. SR, the project PI, provided support to SJ, AK, BK and JK to develop the initial ideas, which were then contributed to by members of the wider team.

8. How were research partners supported to develop writing skills?
The lead authors of this study (SJ and AK - joint lead authors) are amongst the most junior members of the project team: both are Research Associates and neither, at this stage, has studied for a PhD. They were supported initially by SR and subsequently by the whole authorship team to plan the structure of the paper; and via iterative rounds of comments and contributions to a series of drafts.

9. How will research products be shared to address local needs?
This article will be published open access (as required by the funder) and promoted through our networks in Nepal. In addition, the high-income country academics are continuing to work with the NGO partner to implement the recommendations of the paper in relation to further training.

10. How is the leadership, contribution and ownership of this work by LMIC researchers recognised within the authorship?
The order of authors is presented according to their contribution to this paper (and not to seniority, or contribution to the wider project). SJ and AK led on developing the manuscript, with support from SR. Other members of the writing team contributed to drafts and to refining the manuscript. Of the 12 authors, 7 are Nepali (although one of those is based in a high-income country).

11. How have early career researchers across the partnership been included within the authorship team?
We have included early career researchers (SJ, AK, BK, SB, BR) within the authorship team. They all contributed to the design of the project, the fieldwork, and to authoring the manuscript (which was led by SJ and AK).

12. How has gender balance been addressed within the authorship?
Eight authors are male (SJ, AK, SR, BK, JK, MB, TC, DJG, AL) and four authors female (AK, SB, BR, GP). This reflects regrettable gender inequalities in both the UK and Nepal research communities of which we are aware, and are committed to taking steps to address.

For peer review only

13. How has the project contributed to training of LMIC researchers?

Five of the authors (SJ, AK, BK, SB, BR) are junior (pre-PhD) researchers whose salaries were (in part) funded by this project. Through this project, they have all received support and advice in relation to all stages of the research process (project design; ethics; fieldwork design and implementation; analysis; manuscript writing).

14. How has the project contributed to improvements in local infrastructure?

This project has not directly contributed to improvements in local infrastructure.

15. What safeguarding procedures were used to protect local study participants and researchers?

Primary data collection involved 'professional' participants (health workers). All interviews were conducted by two researchers, including at least one female researcher. In-person interviews were conducted in a public place in the context of a training event being run by the NGO partner. Re-interviews were conducted by telephone.

For peer review only

SPQR Checklist

**Topic**	**Line no(s)**
**Title and Abstract**	
Title	1-3
Abstract	5-32
**Introduction**	
Problem formulation	90-135
Purpose or research question	131-135
**Methods**	
Qualitative approach and research paradigm	139-141
Researcher Characteristics and reflexivity	See Reflexivity statement
Context	90-135
Sampling Strategy	153-157
Ethical issues pertaining to human subjects	220-222
Data collection methods	166-207
Data collection instruments and technologies	175-179; 186-198
Units of study	158-162
Data processing	204-207
Data analysis	209-218
Techniques to enhance trustworthiness	204-207; 210-218
**Results/Findings**	
Synthesis and interpretation	224-375
Links to empirical data	224-375
**Discussion**	
Integration with prior work, implications, transferability and contribution(s) to the field	377-435
Limitations	437-451
**Other**	
Conflicts of interest	577-579
Funding	582-584

For peer review only

Gender and caste inequalities in primary healthcare utilization by under-5 children in rural Nepal: an iterative qualitative study into provider perspectives and the potential role of implicit bias

Journal:	*BMJ Open*
Manuscript ID	bmjopen-2022-069060.R1
Article Type:	Original research
Date Submitted by the Author:	04-May-2023

Complete List of Authors:	Joshi, Saugat ; PHASE Nepal<br>Karki, Alisha; PHASE Nepal<br>Rushton, Simon; The University of Sheffield, Department of Politics and International Relations<br>Koirala, Bikash; PHASE Nepal<br>Basnet, Srijana; PHASE Nepal<br>Rijal, Barsha; PHASE Nepal<br>Karki, Jiban; Liverpool School of Tropical Medicine<br>Pohl, Gerda; PHASE Nepal<br>Baidya, Manish; PHASE Nepal<br>Chater, Tim; University of Liverpool<br>Green, Dan; Aston University<br>Lee, Andrew; The University of Sheffield, School of Health and Related Research

**Primary Subject Heading**:	Health services research
Secondary Subject Heading:	Global health
Keywords:	Health policy < HEALTH SERVICES ADMINISTRATION & MANAGEMENT, Quality in health care < HEALTH SERVICES ADMINISTRATION & MANAGEMENT, International health services < HEALTH SERVICES ADMINISTRATION & MANAGEMENT, EDUCATION & TRAINING (see Medical Education & Training)

SCHOLARONE™
Manuscripts

I, the Submitting Author has the right to grant and does grant on behalf of all authors of the Work (as defined in the below author licence), an exclusive licence and/or a non-exclusive licence for contributions from authors who are: i) UK Crown employees; ii) where BMJ has agreed a CC-BY licence shall apply, and/or iii) in accordance with the terms applicable for US Federal Government officers or employees acting as part of their official duties; on a worldwide, perpetual, irrevocable, royalty-free basis to BMJ Publishing Group Ltd ("BMJ") its licensees and where the relevant Journal is co-owned by BMJ to the co-owners of the Journal, to publish the Work in this journal and any other BMJ products and to exploit all rights, as set out in our licence.

The Submitting Author accepts and understands that any supply made under these terms is made by BMJ to the Submitting Author unless you are acting as an employee on behalf of your employer or a postgraduate student of an affiliated institution which is paying any applicable article publishing charge ("APC") for Open Access articles. Where the Submitting Author wishes to make the Work available on an Open Access basis (and intends to pay the relevant APC), the terms of reuse of such Open Access shall be governed by a Creative Commons licence – details of these licences and which Creative Commons licence will apply to this Work are set out in our licence referred to above.

Other than as permitted in any relevant BMJ Author's Self Archiving Policies, I confirm this Work has not been accepted for publication elsewhere, is not being considered for publication elsewhere and does not duplicate material already published. I confirm all authors consent to publication of this Work and authorise the granting of this licence.

For peer review only

Gender and caste inequalities in primary healthcare utilization by under-5 children in rural Nepal: an iterative qualitative study into provider perspectives and the potential role of implicit bias

Corresponding author:

Professor Simon Rushton,

Department of Politics & International Relations, University of Sheffield, Elmfield, Northumberland Road, Sheffield S10 2TU, UK.

Email: simon.rushton@sheffield.ac.uk

Tel: 0114 2221710

Full author list:

Saugat Joshi, PHASE Nepal, Kathmandu, Nepal.

Alisha Karki, PHASE Nepal, Kathmandu, Nepal.

Simon Rushton*, Department of Politics & International Relations, University of Sheffield, Sheffield, UK.

Bikash Koirala, PHASE Nepal, Kathmandu, Nepal.

Srijana Basnet, PHASE Nepal, Kathmandu, Nepal.

Barsha Rijal, PHASE Nepal, Kathmandu, Nepal.

Jiban Karki, Liverpool School of Tropical Medicine, Liverpool, UK.

Gerda Pohl, PHASE Nepal, Kathmandu, Nepal.

Manish Baidya, PHASE Nepal, Kathmandu, Nepal.

Tim Chater, Liverpool Clinical Trials Centre, University of Liverpool, Liverpool, UK.

Dan J Green, Department of Optometry, Aston University, Birmingham, UK.

Andrew Lee, School of Health & Related Research, University of Sheffield, Sheffield, UK.

Keywords: Child Health, Health Services Research, Nepal, implicit bias, unconscious bias, primary health care utilisation, inequalities in primary health care

Word Count: 4888

For peer review only - http://bmjopen.bmj.com/site/about/guidelines.xhtml

Gender and caste inequalities in primary healthcare utilization by under-5 children in rural Nepal: an iterative qualitative study into provider perspectives and the potential role of implicit bias

Abstract

Objective: This study explored provider perspectives on: i) why inequalities in health service utilisation persist; and ii) their knowledge and understanding of the role of patient experience and implicit bias (also referred to as unconscious bias).

Design: A three stage, iterative qualitative study was conducted involving two rounds of in-depth interviews and a training session with healthcare staff. Interview transcripts were analysed using a reflexive thematic approach in relation to the study's aims.

Setting: Participants were recruited from rural hill districts (Mugu, Humla, Bajura, Gorkha and Sindhupalchok) of Nepal

Participants: Clinical staff from 22 rural health posts

Results: Healthcare providers had high levels of understanding of the cultural, educational and socio-economic factors behind inequalities in healthcare utilisation in their communities. However, there was less knowledge and understanding of the role of patient experience - and no recognition at all of the concept of implicit bias.

For peer review only - http://bmjopen.bmj.com/site/about/guidelines.xhtml

Conclusion: It is highly likely that implicit bias affects provider behaviours in Nepal, just as it does in other countries. However, there is currently not a culture of thinking about the patient experience and how that might impact on future utilisation of health services. Implicit bias training for health students and workers would help create greater awareness of unintended discriminatory behaviours. This in turn may play a part in improving patient experience and future healthcare utilisation, particularly amongst disadvantaged groups.

For peer review only

For peer review only - http://bmjopen.bmj.com/site/about/guidelines.xhtml

Strengths and Limitations of the study

- Existing research on health service utilisation in Nepal has been from the patient perspective: this study examines service providers' perspectives on the factors that cause inequalities in health service utilisation.
- The study produced strong evidence that healthcare providers have a good understanding of the social and economic factors that determine service utilisation, and can point to instances of discriminatory treatment.
- To our knowledge, this is one of the few studies that has investigated the possibility of introducing implicit bias training for health workers in Nepal.
- Participants in the study were health post staff employed by a Nepali NGO in selected districts, and do not necessarily reflect the perspectives of all healthcare providers in all areas of the country.
- This study did not examine the experiences of patients, parents or caregivers who receive services: this would be an important avenue for future research.

For peer review only - http://bmjopen.bmj.com/site/about/guidelines.xhtml

Introduction

The world has made substantial progress in reducing childhood mortality and child survival over the past three decades. Globally, the under-5 mortality rate has decreased by 60%, from 93 deaths per 1000 live births in 1990 to 37 in 2020 [1]. Nevertheless, around 5 million children still die each year, largely from causes that are preventable [1]. The health of young children is especially susceptible to socioeconomic inequalities, which have led to significant differences in childhood morbidity and mortality between and within countries [2].

Among the countries of South Asia, Nepal has the highest reported prevalence of under-5 mortality over the past 15 years [3]. In recognition of this, child health is one of the priority programs of the government, which has targeted reducing under-5 mortality to 28 per 1,000 live births and neonatal mortality to 12 per 1,000 live births by 2030 [4]. As a result of this prioritisation, significant improvements have been made in child health services and outcomes over recent years [5]. Despite this, socio-economic inequalities remain an obstacle to tackling Nepal's continuing high rates of child illness and death [6] . In addition to continuing inequalities in the socio-economic determinants of health, health services are still not effectively reaching all individuals, most commonly those from the low castes, ethnic minorities, people living in remote areas, and other marginalised populations [7]. Deeply-rooted societal, gender, caste/ethnic, regional and wealth inequities have made it difficult to address this situation.

Health care utilisation by children under-5 depends on the health-seeking behaviours of their parents and caregivers. As seen in studies elsewhere in South Asia, parents and caregivers do not always appropriately seek health care services for childhood illness for a variety of reasons, including low levels of parental education [8] [9] [10], a preference for traditional healers [11], and living long distances from a health post [12]. Health-seeking patterns for children have also

For peer review only

been found to be highly gendered in Nepal [13], as in other Asian countries [14] [15]. Delays in care-seeking can lead to illnesses becoming more severe, or in some cases mean that the child does not receive treatment at all.

Stigma is one of the causes of health inequalities [16], and discriminatory attitudes and practices among health service providers can discourage healthcare utilisation by certain groups. For example, although caste-based discrimination is illegal in Nepal, continuing cultural belief in the 'untouchability' of Dalits has been found to limit access to healthcare services [16]. When patients do present at a health facility, studies in other settings have shown that patients' experience can significantly affect future utilisation: if people are dissatisfied with services, or feel discriminated against when using them, they are less likely to return [17] [18] [19]. One study conducted in Nepal found that only one-third (35.9%) of respondents reported receiving good communication from health service providers [20], suggesting that shortcomings in patient experience are widespread. Ensuring a good patient experience for all is therefore an important element of attempts to promote health service utilisation amongst marginalised groups [21].

However, this is an under-researched area in Nepal. Relatively few studies have examined the barriers to healthcare utilisation for under-5s in Nepal. Those studies that do exist have examined socio-economic factors affecting the likelihood of people seeking health services from the perspective of patients [10, 11]. While recognising the importance of this patient perspective, in this study we seek to complement this previous work by: i) adopting a service provider-focussed perspective, to understand their perceptions on the reasons for unequal patterns of health care utilisation; and ii) to explore service providers' perspectives on patient experience and how they think this may relate to service utilisation by different groups.

For peer review only - http://bmjopen.bmj.com/site/about/guidelines.xhtml

This qualitative study builds on the findings of a previous quantitative study in which paper-based patient records from 23 rural health posts were digitised and analysed to understand inequalities in health service utilisation, diagnosis and treatment for under-5 children [22]. The data revealed significant differences by gender, caste and geography. For example, we found that children in more remote districts took longer period to present at a health facility after the onset of symptoms than children in less remote districts. When children were brought to a health facility, those from underprivileged groups (e.g. Madhesi and Dalit) were less likely to be given a correct diagnosis of pneumonia, while males were more likely to receive a correct diagnosis than females, suggesting inequalities in the quality of treatment received. While this previous study revealed these inequalities, it did not explain *why* those differences occurred. In the follow-up study reported here, we conducted in-depth interviews (IDIs) with service providers from those same health posts to explore their perceptions as to why these differences exist in their communities, and subsequently designed and implemented training sessions based on role-play scenarios to increase their knowledge and understanding of patient experience.

Methods

Study design

We applied a qualitative approach in this study, employing IDIs with health workers from rural health posts to explore inequalities in service utilisation, focusing on providers' perceptions of the causal mechanisms within the rural communities in which they work. After the initial round of analysis identified notable gaps in the data around the potential effect of service providers' own attitudes and practices, we designed and implemented training and subsequently conducted

For peer review only - http://bmjopen.bmj.com/site/about/guidelines.xhtml

a second round of IDIs to explore interviewees' awareness and understanding of implicit bias (also referred to as unconscious bias).

Setting

In Nepal, each ward (the smallest political unit) has a government-run health post and community health unit providing primary healthcare and (where necessary) referral on to secondary health facilities. In some cases (as in this study) those facilities are additionally supported by staff employed by NGOs. The participants in this study were current clinical staff members, employed by a Nepali NGO, drawn from 22 of the 23 rural health posts that had been part of the preceding quantitative study (for logistical reasons, it was not possible to interview staff from one health post). We report this study in accordance with the Standards for Reporting Qualitative Research checklist (SRQR).

Research Participants

We initially conducted In-Depth Interviews (IDIs) with one health service provider currently working in each of 22 health posts spread across four provinces of Nepal (Bagmati, Gandaki, Karnali and Sudurpaschim provinces), covering the rural hill districts of Gorkha, Sindhupalchok, Mugu, Humla and Bajura. These particular health posts were selected for both the original quantitative study and this follow-up study as they are currently supported by the NGO partner organisation involved in this study, a national NGO employing a total of 78 staff.

Within each health post, we purposively selected the most experienced member of NGO staff (measured in terms of years of service) in order to get as informed an account as possible of the possible reasons for inequalities in healthcare utilisation. The years of experience ranged from 2

For peer review only - http://bmjopen.bmj.com/site/about/guidelines.xhtml

to 14 years. Interviewees included Auxiliary Nursing Midwives (ANMs), Staff Nurses, and Paramedics. The majority of interviewees had experience of having worked in multiple rural health posts. 19 of the interviewees were female and 3 male, with ages ranging from 25 to 35 years. Further characteristics of the interviewees and the associated participant codes are presented in Table 1.

Table 1: Characteristics of interviewees

Participant code	Ethnicity	Education qualification
KII_F_01	Janajati	Intermediate
KII_F_02	Brahmin/Chhetri	Staff Nurse
KII_F_03	Brahmin/Chhetri	Intermediate
KII_F_04	Brahmin/Chhetri	ANM
KII_F_05	Brahmin/Chettri	ANM
KII_F_06	Dalit	ANM
KII_M_07	Brahmin/Chhetri	ANM
KII_F_08	Dalit	ANM
KII_F_09	Brahmin/Chhetri	Staff Nurse
KII_M_10	Brahmin/Chhetri	HA
KII_F_11	Dalit	HA
KII_F_12	Brahmin/Chhetri	ANM
KII_F_13	Brahmin/Chhetri	ANM
KII_F_14	Janajati	ANM
KII_F_15	Brahmin/Chhetri	ANM

For peer review only - http://bmjopen.bmj.com/site/about/guidelines.xhtml

KII_F_16	Janajati	Staff Nurse
KII_F_17	Janajati	ANM/Intermediate pass
KII_M_18	Brahmin/Chhetri	Health Assistant
KII_F_19	Dalit	ANM
KII_F_20	Brahmin/Chhetri	ANM/10+2
KII_F_21	Brahmin/Chhetri	ANM/ 10+2
KII_F_22	Janajati	ANM

Data collection and management

Data collection was via a three-stage iterative process in which the research plans were adapted at each stage in response to initial findings.

The initial round of interviews was undertaken during a week of training events attended by all health staff of the project's NGO partner (which was why only NGO staff, and not government staff, participated). Prior to the IDIs, the results of the preceding quantitative study (i.e. the data on gender, geographic and caste inequalities in these specific health posts) were presented to all of the NGO's staff. The selected participants were then interviewed individually to ascertain their perspectives on *why* these inequalities persist.

The semi-structured interview guide (see Supplemental File 1) was developed based on our previous quantitative study, expert input from medical practitioners, major themes from existing literature and discussion between the members of the research team. The interview guide was initially developed in English and then translated into Nepali. We conducted four interviews as a

For peer review only

pre-test and made necessary changes to the interview schedule to improve the clarity of questions. We then conducted the 18 remaining IDIs.

A rapid analysis of the emerging findings from this first round of 22 interviews was discussed by the members of the research team, and omissions/notable gaps in the data were considered [23], taking into account the published evidence on health service utilisation (in Nepal and elsewhere). During this analysis, the potential role of negative patient experience and implicit bias leading to suboptimal patient experience – even where providers themselves had the best of intentions - was identified as a notable 'silence' in the data.

In the second stage, in response to these emerging findings, a training session on patient experience was designed, drawing on input from team members who had been involved in similar training sessions in other countries. The training session was held two days after the final interview. It included a role play session conducted in pairs in which half of the participants were asked to play the role of a patient bringing a baby to a health post, and the other half to play the role of the service provider. After the role-play consultation was complete, the participants swapped roles. This was followed by a guided de-brief plenary discussion in which patient experience was discussed, first in pairs and later as a whole group, and participants' perspectives on necessary elements of a positive patient experience were collated. This session was designed to prompt reflection amongst participants on patient experience.

In the third stage (2 weeks after the training event had finished and participants had returned to their health posts), a new interview guide was developed focusing on the themes of patient experience and participants' knowledge and understanding of implicit bias. Based on this guide, a second round of follow-up interviews was conducted, via telephone, with all 22 study participants.

For peer review only - http://bmjopen.bmj.com/site/about/guidelines.xhtml

All interviews took place in April and May 2022, carried out by five experienced qualitative researchers (AK, SJ, BR, SB and BK). The training session additionally involved facilitation by JK, GP and SR. We obtained written consent from participants. Both the face-to-face interviews and the telephone interviews were audio recorded with the participants' consent. The average length of the interviews was 25 minutes.

We first transcribed the recordings verbatim into Nepali, and then translated these transcriptions into English. All personal identifiers were replaced with unique codes (see Table 1). Seven of the interview transcripts were back translated to ensure that no meaning was lost during the translation process.

Data analysis methods

Two researchers (AK and SJ) coded the interview transcripts using a reflexive thematic approach in NVivo 12 (Version 12 pro, QRS International) qualitative data management software [24]. The researchers read the full transcripts before starting the coding process. Following this, we organised all the text from the interviews by coding and grouping them into appropriate themes, supported by discussion amongst the members of the research team. During the process, we developed initial sets of codes while thoroughly reading the text followed by combining and modifying them, thus developing a more refined codebook. Finally, we grouped the codes into major thematic categories and the whole research team finalised the codebook for the data analysis process.

Ethical considerations

For peer review only - http://bmjopen.bmj.com/site/about/guidelines.xhtml

Ethical approval for the study was obtained from (Anonymised for peer review) and the Ethical Review Board of the Nepal Health Research Council (NHRC) on 13 March 2020, with Ref No: 1936, ERB Protocol No. 12/2020 P.

Patient and Public Involvement

None

Findings

First round of interviews: Provider explanations for differential utilisation of health services

In this section, we present the explanations given by service providers in the first round of interviews for the observed differences in health service utilisation in the areas in which they work according to i) caste and ii) gender.

i) Caste

Lack of knowledge and education

Most interviewees (n=16) indicated that, in their experience, people from the lower castes lack awareness, knowledge and education on the causes, symptoms, and treatments for diseases.

For peer review only

"When a Brahmin child gets sick, mostly the parents bring them to the health post as soon as possible. But people belonging to the Dalit caste are not educated or aware enough to make such informed decisions. KII_F_08

"People of lower caste are uneducated and are unaware of their health. Janajati people make the children drink local alcohol to treat their condition". KII_F_22

Economic factors

The majority of interviewees (n=16) noted that, in comparison to people from other castes, Dalits tend to be poorer.

The economic situation of Dalits is also poor compared to other groups, which is why they tend to seek household remedies to cure the illnesses of their children". KII_F_08

Although services at health posts are provided free of charge, interviewees explained that economic deprivation could nevertheless affect service utilisation: those from lower castes have less flexibility with their time, often having to work long hours in their own fields and/or for other people to make a living. As a result, they are not able to visit the health post without incurring financial losses, and thus tend not to bring their children for treatment in the early stage of illness.

Preferential use of traditional healers

For peer review only - http://bmjopen.bmj.com/site/about/guidelines.xhtml

Most interviewees (n=18) reported that traditional healers' services are often utilised at the community level, either because they are preferred by patients, or because they are more conveniently located.

> *"In rural areas, faith healers are still the first choice for the majority of people. People who live near health posts try to bring their children as soon as possible, but people who live far from the village centre first try traditional remedies and consume medicinal herbs".* KII_F_08

> *"People who live far from the health post prefer to take their children to a faith healer to treat their condition. They only come to a health facility if they are referred by the faith healer, or if the faith healer is not able to treat their condition".* KII_F_06

Some interviewees (n=8) reported that older people and those who are less educated (often from lower castes) are particularly likely to believe in gods and goddesses and prefer to participate in rituals and make offerings in case of illness. These groups were seen as more likely to take their children to traditional healers, and health education efforts were seen as having had little impact so far:

> *"We are involved in conducting household programs to raise the awareness level and educate people about the importance of their children's health. We ask them to bring their children [to the health post] as soon as they get sick. However, people in the village are still following outdated practices that were taught by their ancestors."* KII_F_10

Deep rooted (and internalised) stigma

For peer review only - http://bmjopen.bmj.com/site/about/guidelines.xhtml

The deep-rooted stigma and discrimination against Dalits in the community was seen as reducing their willingness to bring their children to the health post, resulting in self-exclusion from services. When they do bring their children to the health post, interviewees reported that other patients (for example, those from the Brahmin/Chhetri caste) insist that their children should be treated first. Some interviewees (n=5) reported that many Dalits themselves believe that it is a sin if they touch an upper caste person, so even when they do go to the health post, they sit in a corner, away from other waiting patients. One of our participants said that:

> *"When we demonstrated how 'jaulo' [a kind of porridge] is made, we invited all the people from different castes. When they came, all the so-called upper caste people sat on one side and the lower-class people sat on the other side. While eating food, they did not touch each other. It's not like people set a rule about such things, but the culture was such that a Dalit person believes that it is not nice to touch a person of upper caste".*
> KII_F_06

ii) Gender

Differential attitudes towards male and female children

Providers believed that parents are more likely to take sons to health facilities quickly when they are sick, and to spend more money on their care than they do for their daughters. They reported that parents of daughters are less likely to seek care outside of home and, even when seeking care, they are more likely to go to an informal or traditional healer. Even mothers were seen as being more vigilant about the health of their sons.

For peer review only - http://bmjopen.bmj.com/site/about/guidelines.xhtml

"There was a woman in Bajura district who had three daughters. One of her daughters had severe pneumonia, but she did not bring her to the health post. She said that it would not matter if one daughter dies because she was still of childbearing age and could give birth to another one if this one dies" KII_F_03

"In the community I worked in, there was a woman who had a son after four daughters. She didn't bring any of her daughters when they got ill. But she used to get worried and concerned and brought her son if he suffered even a minor cold. She used to say that after waiting and praying for a long time, they were able to have a son. She brought her son as if it was a very serious case, but it was normal" KII_F_02

Service providers attributed these differences in health-seeking behaviours to deeply-rooted cultural norms around the relative value of male and female children, and their expected future roles and contribution to the family.

"A girl child doesn't even get a naming ceremony. A mother gives a random name based on what she likes". KII_F_08

"People say that a daughter is not going to earn and she will not achieve anything big in life, so they tend to deprive a female child of education as well as not seeking health services. They say that there is no point in educating a daughter since she will get married after a certain age." KII_F_06

"People believe that a male child can carry the legacy of their parents forward. Sometimes, when we are not able to treat their daughters, we refer them to a hospital. When we do so, they ask us if we can treat her or not. When we say no, then they don't

For peer review only - http://bmjopen.bmj.com/site/about/guidelines.xhtml

think it is worth taking her to the hospital. Even if the daughter dies, the mother does not feel guilty." KII_F_06

Second round of interviews: Interviewees' knowledge and understanding of patient experience and implicit bias

As described above, the first round of interviews revealed that interviewees had a very good awareness of the ways in which caste and gender could affect health service utilisation in the communities in which they were working. They did not, however, identify the potential role of provider-side attitudes and behaviours. The training session on patient experience and the follow-up interviews explored these themes in depth. In the second round of interviews, we examined interviewees' experience of discriminatory behaviours, and their knowledge and understanding of the potential impact of implicit bias on their own treatment of patients.

Experiences of unequal patient experience

When asked about the respective experiences that different groups may have when visiting a health post, many interviewees (n=14) were able to give examples of having witnessed colleagues not treating every patient equally.

"It is not so common but, in some cases, health personnel tell a Dalit patient to keep their distance and ask them to do things on their own, just to avoid touching them as they are considered untouchables." KII_F_08

For peer review only

For peer review only - http://bmjopen.bmj.com/site/about/guidelines.xhtml

365 *"When I was working in a remote area of Gorkha district, Dalit children were not*
366 *allowed to sit with other patients and they received their check-up only after Brahmin*
367 *and Chhetri children had been seen"*. KII_F_16

368 *"People of certain castes were not given the respect they deserve and staff [government*
369 *health workers] mostly tried to ignore them as much as possible, just to avoid touching*
370 *them. When we [NGO staff] tried to touch them to treat them, we were told to not to*
371 *touch such patients and to avoid physical contact"* KII_F_09

372 *"As most of the [government] staff working in the health facility are local, they treat*
373 *patients based on their caste/ethnicity. People who are from the upper castes are treated*
374 *with utmost care and respect and those who belong to lower castes are neglected and,*
375 *sometimes, mistreated as well"*. KII_F_09

376 In all cases, such discriminatory treatment was attributed to others - and in all cases to
377 government health staff rather than staff of the NGO (see Limitations).

378 Although, when asked in the training session and interviews, participants agreed with the logic
379 that patient experience could have an impact on whether or not patients chose to return to the
380 health post in case of future illness amongst their children, most indicated that there was no
381 effective feedback mechanism in the health facilities where they work to capture patients'
382 (dis)satisfaction with their experience.

383 *"We know that from their body language. We don't have any specific mechanism, but*
384 *whenever a patient tells us then we get to know about their experiences"*. KII_F_02

For peer review only - http://bmjopen.bmj.com/site/about/guidelines.xhtml

Furthermore, interviewees said that they had not previously received training relating to patient experience. Reflecting on the role play-based training session provided to participants by the research team, one reflected that

"Previously, we used to see things from the side of a health service provider, but this roleplay session made us realise that we need to treat patients with patience. We do not have much patience as we have to see many patients in a day. I think [this training] will definitely support us in our work in the future." KII_F_05

Knowledge and understanding of implicit bias

Although discriminatory treatment was recognised (and always attributed to others), all interviewees (n=22) reported that they had never heard about the concepts of unconscious or implicit bias, nor received any training on it. For example,

"I don't think I have heard about such a term. We don't intentionally try to be biased towards a particular individual. However, there might be some cases that we are not aware of" KII_F_09

"I don't think I have heard or had such an experience about implicit bias in my professional life" KII_F_07

"I have not received any specific training on that. In other training that we received, we are only taught about treating the patient right but we are not aware of the bias related training that you mentioned."KII_F_04

For peer review only - http://bmjopen.bmj.com/site/about/guidelines.xhtml

Discussion

We found that rural healthcare providers had a high level of awareness and understanding of the cultural, educational, and socio-economic factors behind inequalities in healthcare utilisation. The lack of awareness, knowledge and education amongst Dalits and other low castes, their preference for traditional healers, deep-rooted and internalised stigma, and differential parental attitudes towards male and female children, were all commonly identified, and were in accordance with findings in the existing literature from Nepal [6, 7, 10, 11, 13, 25, 26] and elsewhere in South Asia [8-10, 12, 15, 16, 27, 28]

However, participants tended to have far low levels of awareness, understanding and training on the ways in which patient experience might adversely impact service utilisation. Although many respondents reported witnessing discriminatory behaviour by other health service providers, they generally believed that they treat everyone equally in their own practice (see 'Limitations' for our reflections on self-serving bias here). Studies from other countries have demonstrated that poor (and especially discriminatory) patient experience can deter future health service utilization, as well as affect treatment adherence [17-19, 21, 29-31]. However, our findings suggest that in Nepal there is not currently a culture of thinking about patient experience. Neither is there awareness amongst frontline health staff of the concept of implicit bias.

Most previous studies on the role of implicit bias have taken place in the Global North. For example, a narrative review indicated that healthcare providers in the United States show implicit negative attitudes and stereotypes about several stigmatised groups [32]. Another study reported that the majority of health professionals showed preferential treatment towards non-Hispanic white children or adults over American Indian patients [33]. These studies suggest that being a child does not protect one from being on the receiving end of implicit bias. Although we did not

For peer review only - http://bmjopen.bmj.com/site/about/guidelines.xhtml

utilise methods that could have demonstrated attitudes or behaviours associated with implicit bias amongst our participants, the existing literature from elsewhere suggests that it is extremely unlikely that our interviewees are completely free from such biases. The fact that implicit bias is frequently unrecognised by those who hold such biases is well known. Numerous studies have shown that self-reporting of implicit bias is not reliable as it is challenging for individuals to critically evaluate their own thoughts [34, 35]. Such biases exist in everyone, even well-intentioned healthcare workers (and also, of course, the members of this research team). Evidence indicates that healthcare professionals have the same levels of implicit bias as the general public [36].

Healthcare providers' awareness of implicit bias is not well documented in the context of Nepal and our literature review revealed very few previous studies in Nepal gave this topic even a passing mention [37] (for an exception see [38]). However, it is evident that implicit bias is significantly related to patient-provider interactions, treatment decisions, and patient health outcomes in all contexts [33], and certainly will be in Nepal. All of this leads us to suspect that bias amongst our participants is currently unrecognised rather than absent.

This study has revealed a need for further research on implicit bias in healthcare settings in Nepal. Whilst the literature from other countries suggests that it is highly likely that implicit bias impacts on patient treatment in Nepal, it is important to understand the extent, consequences and drivers of this bias in the particular social context of Nepal - as well as to explore effective ways to counter this.

Our findings also have implications for practice as implicit bias has the potential to affect both treatment outcomes, and also whether patients (especially those from disadvantaged groups) bring their children to health facilities for treatment in future. Studies from other countries have

For peer review only - http://bmjopen.bmj.com/site/about/guidelines.xhtml

shown that the provision of training to increase the awareness of individuals about their own biases can have a positive impact, equipping them to better manage and account for these biases in future [39]. Many different approaches to such training are available. To take one example, the Implicit Association Test (IAT), created in 1998, is the most widely used computerised online tool for measuring and bringing awareness of implicit bias [40]. Neither the IAT nor any of the myriad other forms of training and awareness-raising about implicit bias have, to our knowledge, been widely utilised in Nepal – including amongst healthcare workers. Although training cannot by itself completely remove bias, confronting health service providers with evidence of their biases and providing training on how to counter these biases is a necessary first step [32, 40]. We would recommend that Nepalese health/medical schools, the government of Nepal and other employers of health service providers in the country investigate the possibility of introducing implicit bias training.

Conclusion

The impact that provider-side factors may have on health service utilisation in Nepal has not been widely studied, and this is, to our knowledge, the first paper to explore service providers' perspectives on the potential roles of patient experience and implicit bias in deterring health post utilisation for young children.

In some ways, our findings make uncomfortable reading for the NGO partner that participated in this study. However, we would argue that there is a more positive way of interpreting these findings. Given what is known about the difficulties of relying on self-reported biases, implicit bias is likely to be an issue across all health workers in Nepal and not specific to this particular

For peer review only - http://bmjopen.bmj.com/site/about/guidelines.xhtml

NGO. This NGO is the first in Nepal, to our knowledge, to properly investigate this issue. Following on from the training provided in patient experience, the next step in this process - in line with our recommendation above - would be for the NGO to pursue the wider use of validated implicit bias training with staff, in order to provide them with the practical awareness and skills to recognize and mitigate their own biases as well as their unintentional involvement in the perpetuation of discrimination.

More widely in Nepal, implicit bias training (in health/medical schools and for government and non-government health staff) would be a first step in creating greater awareness about unintended discriminatory behaviours, improving patient experience as well as future healthcare utilisation amongst disadvantaged groups. Policy makers and curriculum designers should introduce such training in order to minimize the impact of implicit bias in order to improve patient experience and, in turn, encourage greater utilisation of health facilities amongst marginalised groups.

Competing interests statement

Authors SJ, AK, BK, SB, BR, JK, GP and MB are either currently or previously employed by PHASE Nepal, the NGO partner for this study and the employer of the study participants.

Funding statement

This research was supported by the UK Economic and Social Research Council's GCRF NGO Secondary Data Analysis Initiative, Grant Ref: ES/T010436/1.

For peer review only - http://bmjopen.bmj.com/site/about/guidelines.xhtml

Data Availability statement

Anonymised data are available upon request.

Contribution Statement

SR, JK, GP, MB, TC, DJG and AL all contributed to the planning of the wider project of which this is part.

SJ, AK, SR, BK, CB, BR, JK and GP contributed to the planning and design of the qualitative component of the study which is reported in this manuscript.

SJ, AK, BK, SB and BR conducted the fieldwork.

SJ, AK, SR and JK undertook the initial analysis after the first round of interviews.

AK, SJ, SB, BR, BK, SR, JK and GP conducted the subsequent analysis.

SJ and AK, with support from SR, BK and JK, led on writing the first draft of the manuscript.

All authors contributed to the final version of the manuscript.

SR submitted the study and has overall responsibility for the overall content.

References:

1. WHO. *Child mortality (under 5 years)*. 2022 [cited 2022 26 May]; Available from: https://www.who.int/news-room/fact-sheets/detail/levels-and-trends-in-child-under-5-mortality-in-2020.
2. Barros, F.C., et al., *Socioeconomic inequities in the health and nutrition of children in low/middle income countries.* Revista de Saúde Pública, 2010. **44**: p. 1-16.
3. Sohail, H. and S. Neupane, *Prevalence of and factors associated with under-5 mortality in South Asia.* International Health, 2018. **11**(2): p. 119-127.
4. *National Planning Commission (Nepal). Nepal's Sustainable Development Goals, Baseline Report, 2017. Kathmandu, Nepal: Government of Nepal/National Planning Commission.* 2017.
5. *Ministry of Health. Nepal's every newborn action plan (2016), Kathmandu*. 2016; Available from: https://www.healthynewbornnetwork.org/hnn-content/uploads/NENAP-final-lowresolution.pdf.

For peer review only - http://bmjopen.bmj.com/site/about/guidelines.xhtml

6. Pandey, J.P., et al., *Maternal and child health in Nepal: The effects of caste, ethnicity, and regional identity*, in *DHS Further Analysis Reports No. 73*. 2013, ICF International: Calverton, Maryland, USA.
7. Ghimire, U., et al., *Inequalities in health outcomes and access to services by caste/ethnicity, province, and wealth quintile in Nepal*, in *DHS Further Analysis Reports No. 117*. 2019, ICF: Rockville, Maryland, USA.
8. Kachoria, A.G., et al., *The association of religion with maternal and child health outcomes in South Asian countries.* Plos one, 2022. **17**(7): p. e0271165.
9. khanam, M. and E. Hasan, *Inequalities in health care utilization for common illnesses among under five children in Bangladesh.* BMC Pediatrics, 2020. **20**(1): p. 192.
10. Sreeramareddy, C.T., et al., *Care seeking behaviour for childhood illness-a questionnaire survey in western Nepal.* BMC international health and human rights, 2006. **6**(1): p. 1-10.
11. Pokhrel, S. and R. Sauerborn, *Household decision-making on child health care in developing countries: the case of Nepal.* Health policy and planning, 2004. **19**(4): p. 218-233.
12. Sreeramareddy, C.T., T. Sathyanarayana, and H.H. Kumar, *Utilization of health care services for childhood morbidity and associated factors in India: a national cross-sectional household survey.* PLoS one, 2012. **7**(12): p. e51904.
13. Pokhrel, S., et al., *Gender role and child health care utilization in Nepal.* Health policy, 2005. **74**(1): p. 100-109.
14. Yan, H.X. and Q. Ren, *Selective neglect: Gender disparities in children's healthcare utilization in rural China.* Chinese journal of sociology, 2019. **5**(3): p. 283-311.
15. Ismail, S.A., et al., *Gender-related differences in care-seeking behaviour for newborns: a systematic review of the evidence in South Asia.* BMJ global health, 2019. **4**(3): p. e001309.
16. Thapa, R., et al., *Caste exclusion and health discrimination in South Asia: A systematic review.* Asia Pacific Journal of Public Health, 2021. **33**(8): p. 828-838.
17. Chapman, E.N., A. Kaatz, and M. Carnes, *Physicians and implicit bias: how doctors may unwittingly perpetuate health care disparities.* Journal of general internal medicine, 2013. **28**: p. 1504-1510.
18. Narayan, M.C., *CE: Addressing implicit bias in nursing: A review.* AJN The American Journal of Nursing, 2019. **119**(7): p. 36-43.
19. Kelley, J.M., et al., *The influence of the patient-clinician relationship on healthcare outcomes: a systematic review and meta-analysis of randomized controlled trials.* PloS one, 2014. **9**(4): p. e94207.
20. Trimmer, C. and M. Målqvist, *Clinical communication and caregivers' satisfaction with child healthcare in Nepal; results from Nepal Health Facility Survey 2015.* BMC health services research, 2019. **19**: p. 1-8.
21. Baidya, M., V. Gopichandran, and K. Kosalram, *Patient-physician trust among adults of rural Tamil Nadu: a community-based survey.* Journal of Postgraduate Medicine, 2014. **60**(1): p. 21.
22. Shrestha, B., et al., *Determinants affecting utilisation of health services and treatment for children under-5 in rural Nepali health centres: a cross-sectional study.* BMC Public Health, 2022. **22**(1): p. 1-15.
23. Poland, B. and A. Pederson, *Reading between the lines: Interpreting silences in qualitative research.* Qualitative inquiry, 1998. **4**(2): p. 293-312.
24. Hilal, A.H. and S.S. Alabri, *Using NVivo for data analysis in qualitative research.* International interdisciplinary journal of education, 2013. **2**(2): p. 181-186.
25. French, A.N., *Dalits and mental health: investigating perceptions, stigma and barriers to support in Kathmandu, Nepal.* Journal of Global Health Reports, 2020. **4**: p. e2020009.

For peer review only

For peer review only - http://bmjopen.bmj.com/site/about/guidelines.xhtml

26. Nepali, K.B., *Untouchability and Caste Based Discrimination in Nepal.* Year 20, No. 18, June, 2018 A Nepalese Journal of Participatory Development: p. 49.
27. Panezai, S., M.M. Ahmad, and S.E. Saqib, *Factors affecting access to primary health care services in Pakistan: a gender-based analysis.* Development in practice, 2017. **27**(6): p. 813-827.
28. Barcellos, S.H., L.S. Carvalho, and A. Lleras-Muney, *Child gender and parental investments in India: Are boys and girls treated differently?* American Economic Journal: Applied Economics, 2014. **6**(1): p. 157-189.
29. Hausmann, L.R., et al., *Perceived discrimination in health care and health status in a racially diverse sample.* Medical care, 2008: p. 905-914.
30. Lee, C., S.L. Ayers, and J.J. Kronenfeld, *The association between perceived provider discrimination, health care utilization, and health status in racial and ethnic minorities.* Ethnicity & disease, 2009. **19**(3): p. 330.
31. Burgess, D.J., et al., *The association between perceived discrimination and underutilization of needed medical and mental health care in a multi-ethnic community sample.* Journal of Health Care for the Poor and Underserved, 2008. **19**(3): p. 894-911.
32. Zestcott, C.A., I.V. Blair, and J. Stone, *Examining the presence, consequences, and reduction of implicit bias in health care: a narrative review.* Group Processes & Intergroup Relations, 2016. **19**(4): p. 528-542.
33. Hall, W.J., et al., *Implicit racial/ethnic bias among health care professionals and its influence on health care outcomes: a systematic review.* American journal of public health, 2015. **105**(12): p. e60-e76.
34. Greenwald, A.G. and M.R. Banaji, *Implicit social cognition: attitudes, self-esteem, and stereotypes.* Psychological review, 1995. **102**(1): p. 4.
35. Dang, J., K.M. King, and M. Inzlicht, *Why are self-report and behavioral measures weakly correlated?* Trends in cognitive sciences, 2020. **24**(4): p. 267-269.
36. FitzGerald, C. and S. Hurst, *Implicit bias in healthcare professionals: a systematic review.* BMC medical ethics, 2017. **18**(1): p. 1-18.
37. KC, A., et al., *Mistreatment of newborns after childbirth in health facilities in Nepal: results from a prospective cohort observational study.* Plos one, 2021. **16**(2): p. e0246352.
38. Tergesen, C.L., et al., *Impact of service user video presentations on explicit and implicit stigma toward mental illness among medical students in Nepal: a randomized controlled trial.* International journal of environmental research and public health, 2021. **18**(4): p. 2143.
39. Marcelin, J.R., et al., *The impact of unconscious bias in healthcare: how to recognize and mitigate it.* The Journal of infectious diseases, 2019. **220**(Supplement_2): p. S62-S73.
40. Arif, S.A. and J. Schlotfeldt, *Gaps in Measuring and Mitigating Implicit Bias in Healthcare.* Frontiers in Pharmacology, 2021. **12**.

For peer review only - http://bmjopen.bmj.com/site/about/guidelines.xhtml

Gender and caste inequalities in primary healthcare utilization by under-5 children in rural Nepal: an iterative qualitative study into provider perspectives and the potential role of implicit bias

Interview guides

1. First round interviews

1. Could you please introduce yourself?

कृपया तपाइको परिचय दिनुहोस्।

[Name, age, designation, place of work, qualification/education, experience in years (in rural areas), overall work experience)

[नाम , उमेर , पद , शिक्षा/योग्यता , अनुभव(वर्षमा) (ग्रामीण क्षेत्रमा), समग्र कार्य अनुभव]

2. Health seeking behavior (In terms of Gender, Ethnicity and Geography)

स्वास्थ्य सेवा लिन स्वास्थ्य संस्था आउने बानी/ प्रचलन (लिङ्ग, जाती , भूगोल को आधारमा)

2.1 How do people access the health post when they are unwell? What determines whether parents do or do not bring their child, and how quickly?

मानिसहरू अस्वस्थ (बिरामी) हुँदा कसरी स्वास्थ्य संस्था सम्म आइपुग्छन् ? अभिभावकले बच्चालाई स्वास्थ्य संस्था ल्याउने वा नल्याउने कुरा के ले निर्धारण गर्छ र कत्तिको छिटो ल्याउँछन् ?

2.2 Have you noticed any differences between different groups regarding how quickly they bring their child for treatment?

स्वास्थ्य संस्थामा बिरामी बच्चालाई उपचार गर्ने ल्याउने क्रममा कति छिटो ल्याइन्छ भन्नेमा फरक फरक समूहमा भिन्नता पाउनु भएको छ ?

For peer review only

2.3 The research data reflects differences between some groups? How do you think this can be explained?

अनुसन्धानको तथ्याङ्क (डाटा) हरूमा फरक फरक समूहमा भिन्नता देखिएको छ ? यसलाई कसरी व्याख्या गर्न / बुझ्न सकिन्छ ?

2.4 [Possible solutions for discussed issues/ problems]

छलफल भएका समस्याहरूको समाधान के हुन सक्छ होला भनेर सोध्नुहोस्

3. Discrimination during diagnosis and treatment (in terms of Sex, Ethnicity, Geography)

स्वास्थ्य संस्थामा निदान तथा उपचार गर्ने क्रममा हुने भेदभाव (लिङ्ग, जाती , भूगोल को आधारमा)

3.1 What kind of different groups visit this health post/facility?

यो (तपाईँले काम गर्ने) स्वास्थ्य संस्थामा कुन कुन समूह वा समुदायका मानिसहरू आउने गर्छन् ?

3.2 Can you tell us a bit about the skilled health workers in this facility?

यो स्वास्थ्य संस्थामा दक्ष / तालिम प्राप्त स्वास्थ्यकर्मीहरूको बारेमा केही बताइदिनुहुन्छ ?

3.3 Do you think some health workers treat different groups differently?

तपाईंको बिचारमा केही स्वास्थ्यकर्मीहरुले फरक फरक समूह /समुदायका मानिसलाई लाई फरक किसिमले व्यवहार गर्छन् ?

3.4 The research data reflects difference in diagnosis and treatment among different groups? How do you think this can be explained?

अनुसन्धानको तथ्याङ्क (डाटा) ले निदान र उपचारमा फरक फरक समूह बिचमा भिन्नता भएको पाइएको छ । यसलाई कसरी लिनुहुन्छ (कसरी व्याख्या गर्न/बुझ्न सकिन्छ)?

3.5 Knowing that there are differences, how do you think this can be improved?

For peer review only

उपचार र निदानका क्रममा भिन्नताहरू छन् भन्ने जानिसक्दा , तपाइको बिचारमा यसलाई सुधार गर्न के गर्नु पर्छ होला ?

2. Second round interviews

1. Have you previously received any training about patient experience (before the recent training session)?

स्वास्थ्यकर्मीले दिएको सेवाप्रति बिरामीको अनुभवबारे तपाइले कुनै तालिम लिनु भएको छ?

1.1 Could you provide us any example where you have experienced implicit bias in terms of providing health service?

तपाइले दिनुभएको निष्पक्ष सेवाप्रति कुनै बिरामीले नराम्रो अनुभव गर्नुभएको कुनै उदाहरण दिनसक्नु हुन्छ?

1.2 What was your experience of the roleplay session we conducted during our training workshop? What do you think about its usefulness?

बिरामीको भूमिका निर्वाह गरेर गर्नुभएको अभ्यास प्रति तपाइको अनुभव कस्तो रह्यो ? तपाइलाई यो कत्तिको उपयोगी लाग्यो ?

2. Do you ever have conversation among colleagues about whether all patients are being treated the same or not?

के तपाई आफ्नो सहकर्मीसँग बिरामीले पाउने समान व्यवहारबारे छलफल गर्नुहुन्छ ?

3. Are there any ways where they get feedback from their patient about how their experience was?

तपाइले दिनुभएको सेवाप्रति बिरामीको कस्तो प्रतिक्रिया पाउनु भएको छ?

4. Do you have anything to add on this other than what we have discussed?

For peer review only

हामीले छलफल गरेको विषयहरूमा तपाईँलाई केही थप कुरा भन्न मन मन लागेको छ ?

For peer review only

SPQR Checklist

Topic	Line no(s)
**Title and Abstract**	
Title	1-3
Abstract	5-32
**Introduction**	
Problem formulation	90-135
Purpose or research question	131-135
**Methods**	
Qualitative approach and research paradigm	139-141
Researcher Characteristics and reflexivity	See Reflexivity statement
Context	90-135
Sampling Strategy	153-157
Ethical issues pertaining to human subjects	220-222
Data collection methods	166-207
Data collection instruments and technologies	175-179; 186-198
Units of study	158-162
Data processing	204-207
Data analysis	209-218
Techniques to enhance trustworthiness	204-207; 210-218
**Results/Findings**	
Synthesis and interpretation	224-375
Links to empirical data	224-375
**Discussion**	
Integration with prior work, implications, transferability and contribution(s) to the field	377-435
Limitations	437-451
**Other**	
Conflicts of interest	577-579
Funding	582-584

For peer review only
